# A novel tobamo-like mycovirus with filamentous particles replicates in plant cells

**DOI:** 10.1128/jvi.02102-24

**Published:** 2025-03-31

**Authors:** Meilian Hong, Fengjuan Tian, Zhenmei Song, Hongmei Liu, Yigang Tong, Tingting Zhang

**Affiliations:** 1 Engineering Research Center of Health Medicine Biotechnology of Institution of Higher Education of Guizhou Province, School of Biology and Engineering (School of Modern Industry for Health and Medicine)/School of Basic Medical Sciences, Guizhou Medical University 655509, Guiyang, China; 2 Key Laboratory of Infectious Immune and Antibody Engineering of Guizhou Province, Engineering Research Center of Cellular Immunotherapy of Guizhou Province, School of Biology and Engineering (School of Modern Industry for Health and Medicine), Guizhou Medical University 74628, Guiyang, China; 3 Key Laboratory of Microbiology and Parasitology of Education Department of Guizhou, School of Basic Medical Science, Guizhou Medical University 74628, Guiyang, China; 4 Beijing Advanced Innovation Center for Soft Matter Science and Engineering, College of Life Science and Technology, Beijing University of Chemical Technology 627792 , Beijing, China; Iowa State University, Ames, Iowa, USA

**Keywords:** fungal virus, tobamo-like virus, filamentous virus particles, *Nigrospora aurantiaca*

## Abstract

Parasitic and symbiotic plant–fungus relationships have existed for millions of years, and phylogenetic analyses of certain virus families indicate transmission between plants and fungi. A group of tobamo-like viruses from various fungi has recently been identified. Tobamo-like viruses are homologous to plant viruses of the *Tobamovirus* genus in the *Virgaviridae* family, but it was unknown whether they form typical virus particles and can replicate and exhibit cell-to-cell movement in plants. Here, a novel tobamo-like virus, Nigrospora aurantiaca tobamo-like virus 1 (NaTLV1), obtained from the phytopathogenic fungus *Nigrospora aurantiaca*, was characterized. Its genome (10,301 nucleotides) comprises four open reading frames (ORFs) and a poly(A) tail. ORF1 encodes a methyltransferase and helicase, and ORF2 encodes RNA-dependent RNA polymerase, which are closely related to proteins of *Virgaviridae* viruses. ORF3 encodes a putative movement protein, and ORF4 encodes a putative coat protein, which are closely related to proteins of *Gammaflexiviridae* and *Betaflexiviridae* viruses. NaTLV1 formed filamentous virus particles and could replicate in *Nicotiana benthamiana*. However, the cell-to-cell movement of NaTLV1 was not observed in *N. benthamiana* but could not be ruled out. NaTLV1 underwent horizontal and vertical transmissions via hyphal anastomosis and conidia, respectively. There were no significant differences in phenotype or virulence between NaTLV1-infected (A4) and NaTLV1-free (V1) *N. aurantiaca* strains. In general, our findings may be useful in elucidating the origination and evolution of tobamo-like viruses, plant virus families (*Virgaviridae* and *Betaflexiviridae*), and a fungal virus family (*Gammaflexiviridae*).

**IMPORTANCE:**

Cross-kingdom infections involving plant- and fungal-associated viruses have been directly observed in nature, and some of these viruses share a high degree of genetic similarity. A group of novel tobamo-like viruses with nonsegmented single-stranded RNA genomes was recently isolated from diverse fungal groups. Here, we identified a novel virus, Nigrospora aurantiaca tobamo-like virus 1 (NaTLV1), in the phytopathogenic fungus *Nigrospora aurantiaca*. NaTLV1 was phylogenetically related to other tobamo-like viruses, plant-associated viruses in the *Virgaviridae* and *Betaflexiviridae* families, and fungus-associated viruses in the *Gammaflexiviridae* family. NaTLV1 formed filamentous virus particles and could replicate in *Nicotiana benthamiana,* but the cell-to-cell movement of NaTLV1 was not observed. This study provides potential insights into the origins and evolution of mycoviruses originating from plant viruses and indicates that tobamo-like viruses may have adapted to an intracellular lifestyle during evolution. The identification of novel tobamo-like viruses is crucial for understanding fungal and plant virus evolution.

## INTRODUCTION

Plants and fungi have exhibited parasitic and symbiotic interactions for millions of years (1–4). Both plants and fungi are frequently infected by diverse groups of viruses (5–8). Numerous metagenomic studies have demonstrated that viruses belonging to several families can be transmitted between the plant and fungal kingdoms (5, 9–12). For instance, viruses belonging to the *Endornaviridae*, *Totiviridae*, *Chrysoviridae*, *Amalgaviridae*, and *Partitiviridae* families are known to infect both plants and fungi (6, 11–14). Additionally, a previous study has demonstrated that some members of the *Virgaviridae* family may transmit from plants to fungi (15).

Members of the *Virgaviridae* family are currently classified into seven genera (*Furovirus*, *Goravirus*, *Hordeivirus*, *Pecluvirus*, *Pomovirus*, *Tobamovirus*, and *Tobravirus*) according to their genome organization and other features, including the mode of transmission (16, 17). These viruses have a (+) single-stranded (ss) RNA genome of 6.3 kb–13 kb with the rod-shaped virus particles. Additionally, the virus particles are devoid of an envelope and have a diameter and length of approximately 20 and 300 nm, respectively; however, the length can vary, primarily according to the viral genus (16, 17). Unlike the multipartite genomes of the other six genera, the (+)ssRNA linear genomes of viruses in the *Tobamovirus* genus are monopartite (16, 17).

The first complete genome of a tobamo-like virus, Macrophomina phaseolina tobamo-like virus 1 (isolated from a fungal host), was reported in 2016 (15). Since then, at least 10 other tobamo-like viruses (isolated from various fungal hosts) have been recorded in GenBank (Table S1) (18–22). These viruses generally have a (+)ssRNA genome of 9 kb–13 kb, which comprises a short poly(A) tail and three or four open reading frames (ORFs) (15, 18–22). The first two ORFs encode an RNA-dependent RNA polymerase (RdRp), methyltransferase (Mtr), and helicase (Hel), which are closely related to proteins in plant viruses belonging to the *Tobamovirus* genus in the *Virgaviridae* family. However, it remains to be determined whether tobamo-like viruses form typical virus particles, and studies on the infectivity and virulence of these viruses in plants are limited.

Plant- and fungi-infecting viruses display moderate morphotypical diversity closely related to their taxon, evolution, and host, forming bacilliform, icosahedral, or filamentous virus particles (23–26). Filamentous virus particles are characteristic of many (+)ssRNA plant virus families, including *Closteroviridae*, *Potyviridae*, *Alphaﬂexiviridae*, and *Betaﬂexiviridae*, a few (+)ssRNA fungal viruses in the *Gammaﬂexiviridae* family, and the unclassified double-stranded RNA fungal virus Colletotrichum camelliae filamentous virus 1 (https://ictv.global/msl) (23, 24).

The fungus *Nigrospora aurantiaca* belongs to the *Nigrospora* genus, which is an important ascomycetes genus. *N. aurantiaca* produces red pigment and many diverse secondary metabolites (27, 28) and is a pathogen of important economic crops, causing various diseases, including leaf spot on tobacco and leaf blight disease of peanuts in China (29, 30). This danger caused by *N. aurantiaca* in China warrants attention. To the best of our knowledge, there are no reports of mycoviral infections in *N. aurantiaca*.

In this study, a novel tobamo-like virus, named Nigrospora aurantiaca tobamo-like virus 1 (NaTLV1), was identified in the phytopathogenic fungus *N. aurantiaca*. Subsequent investigation revealed that NaTLV1 forms filamentous virus particles, and it is capable of replication but not cell-to-cell movement in the model plant *Nicotiana benthamiana*. The phenotypic characteristics of NaTLV1 infection were determined by comparing the NaTLV1-infected (A4) and NaTLV1-free (V1) *N. aurantiaca* strains. However, NaTLV1 infection did not affect the colony morphology, size, and conidiation of *N. aurantiaca*, nor did it affect the virulence of *N. aurantiaca* on *N. benthamiana* leaves. The findings indicate that the novel tobamo-like virus NaTLV1, along with other tobamo-like viruses, likely constitute a new viral genus or family, and they provide novel insights into the evolution of plant and fungal viruses.

## RESULTS

### Sequencing and analysis of the whole genome of NaTLV1 from *N*. *aurantiaca*

**Fig 1:**
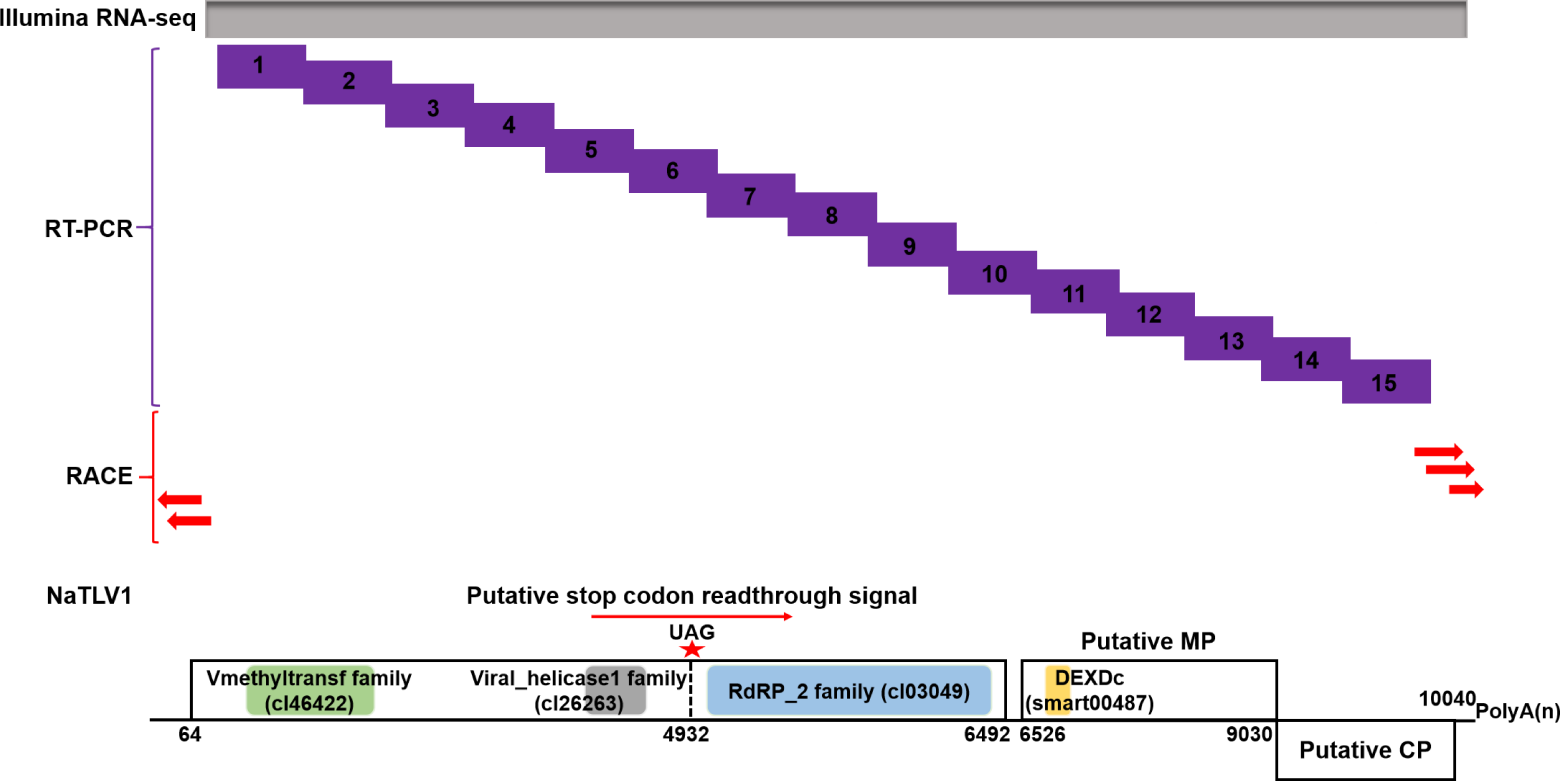
Schematic representation of the NaTLV1 whole-genome sequencing strategy. Gray box represents Illumina RNA sequencing. Purple boxes represent reverse transcription PCR (RT-PCR) followed by Sanger sequencing. Red arrows represent 5´ and 3´ rapid amplification of cDNA ends (RACE) of NaTLV1. The genome organization of NaTLV1 is depicted in the lower panel. Black boxes represent ORFs, while single black lines represent untranslated regions. The conserved Mtr (green), Hel (gray), and RdRp (blue) domain sequences in ORF1 and ORF2 and the conserved DEAD-like helicase superfamily (DEXDc) domain sequences (yellow) in ORF3 are indicated using different background colors.

Agarose gel electrophoresis of dsRNA isolated from mycelial extracts of *N. aurantiaca* strain A4 revealed the presence of a single dsRNA segment that was about 10 K (Fig. S1). The total RNA from *N. aurantiaca* strain A4 was sequenced using the Illumina NovaSeq 6000 (PE150) sequencing platform (Illumina, San Diego, CA, USA) (Fig. 1). A total of 38,825,560 clean reads were obtained and subjected to *de novo* assembly. Only one contig, 10,376 nucleotides (nt) in length, exhibited homology to the viral sequence of Acidomyces richmondensis tobamo-like virus 1, named Nigrospora aurantiaca tobamo-like virus 1 (Fig. S2A). Coverage of the reads mapped against the assembled genome was shown in Fig. S2B, with a maximum depth of 5,282, and the average depth of the NaTLV1 was 2,829. Results of BLASTn and BLASTp searches between the longest contig and other fungus-associated tobamo-like viruses were listed (Tables S2 and S3).

Next, we used the primers in Table S4 and the strategy shown in Fig. 1 to further confirm the full-length genome of NaTLV1, which, excluding the poly(A) tail, is 10,224 nt long and has a GC content of 51.7%. The whole-genome sequence of NaTLV1, which consists of a poly(A) tail and four partially overlapping ORFs (Fig. 1), was submitted to GenBank (accession number OR228589.1).

The BLASTx search and conserved domain analysis revealed that ORF1 (nt 64–4,932) encodes a viral Mtr (amino acids [aa] 54–320) and a protein belonging to the viral RNA Hel superfamily (aa 1,328–1,585) (Fig. 1), while ORF2 (nt 4,932–6,492) encodes an RdRp domain (aa 54–499). ORF1 and ORF2 exhibited the highest similarity to the putative replicase readthrough protein (EMR61648.1) of the endogenous Eutypa lata tobamo-like virus (31), but the aa sequence identities were only 37.38% and 52.58%, respectively. The stop codon readthrough mechanism is conserved in the *Virgaviridae* family and commonly occurs between the RNA Hel and RdRp domains (16, 17). A similar in-frame UAG stop codon was identified between the RNA Hel (encoded by ORF1) and RdRp (encoded by ORF2) domains of NaTLV1. We therefore suggest that NaTLV1 ORF1 and ORF2 can be translated via readthrough of the UAG stop codon to generate a fusion protein (Fig. 1).

ORF3 (nt 6,526–9,030) encodes a putative movement protein (MP) of 91.9 kDa which contains a DEAD-like helicase superfamily (DEXDc) domain (Fig. 1). It exhibited the highest similarity to the putative MP (AZT88675.1) of Acidomyces richmondensis tobamo-like virus 1.

ORF4 (nt 9,030–10,040) encodes a putative coat protein (CP) of 37.0 kDa (155 aa). It exhibited the highest similarity to a putative CP (QED42913.1) of Mycosphaerella tobamovirus B.

Thus, the sequences of the RdRp domain, putative MP, and putative CP of NaTLV1 exhibited the highest similarity to proteins of unclassified tobamo-like viruses (Table S5).

### Phylogenetic analysis and genomic characterization of NaTLV1

**Fig 2:**
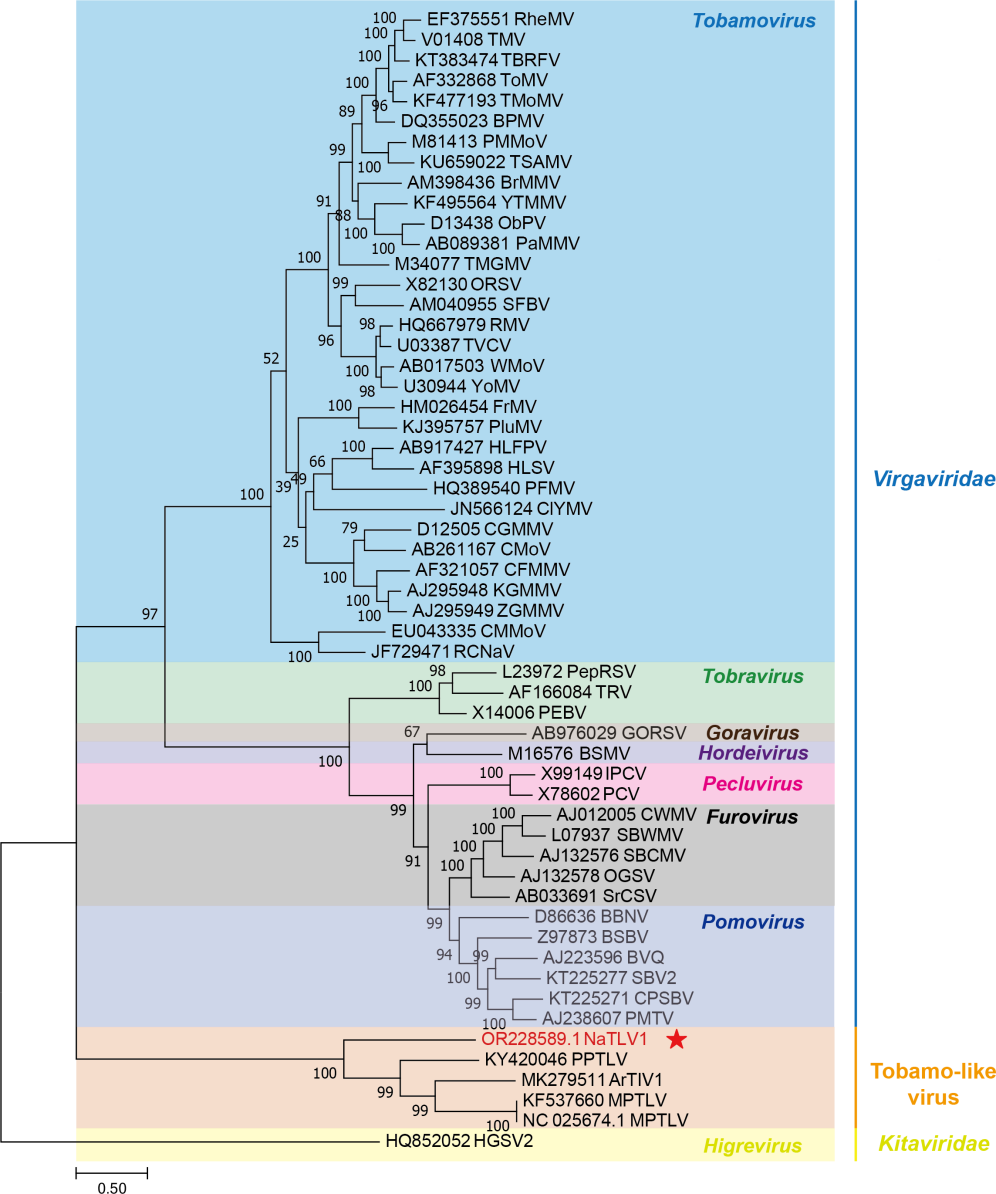
Phylogenetic analysis of RdRp nucleotide (nt) sequences of representative viruses in the *Virgaviridae* family. The phylogenetic tree was estimated using a maximum-likelihood method based on aligned RdRp nt sequences from various genera in the *Virgaviridae* family, which are distinguished by varying background colors in the tree. The NaTLV1 identified in this study is marked with a red star. Outgroups included the nt sequence of RdRp from Hibiscus green spot virus 2 in the *Kitaviridae* family. Branch scale bar = 0.5 substitutions per site.

The phylogenetic tree was constructed based on RdRp nt sequences of NaTLV1, other unclassified tobamo-like viruses, and viruses belonging to the *Virgaviridae* family, which clustered NaTLV1 together with tobamo-like viruses in a single branch, forming a distinct branch within the *Virgaviridae* family (Fig. 2). Therefore, tobamo-like viruses, including NaTLV1, may represent a new genus or family that is closely related to the *Virgaviridae* family.

Interestingly, the putative MPs of NaTLV1 and related tobamo-like viruses exhibited significant differences compared to the MPs of other genera in the *Virgaviridae* family, including significantly longer aa sequences (Table S6). The putative MP of NaTLV1 exhibited no nt sequence homology with any known sequences. It exhibited the highest aa sequence homology with the MP of Acidomyces richmondensis tobamo-like virus 1 (with a coverage of 99% and an identity of 49.07%) but no aa sequence homology with other *Virgaviridae* members (except for tobamo-like viruses) (Table S5).

**Fig 3:**
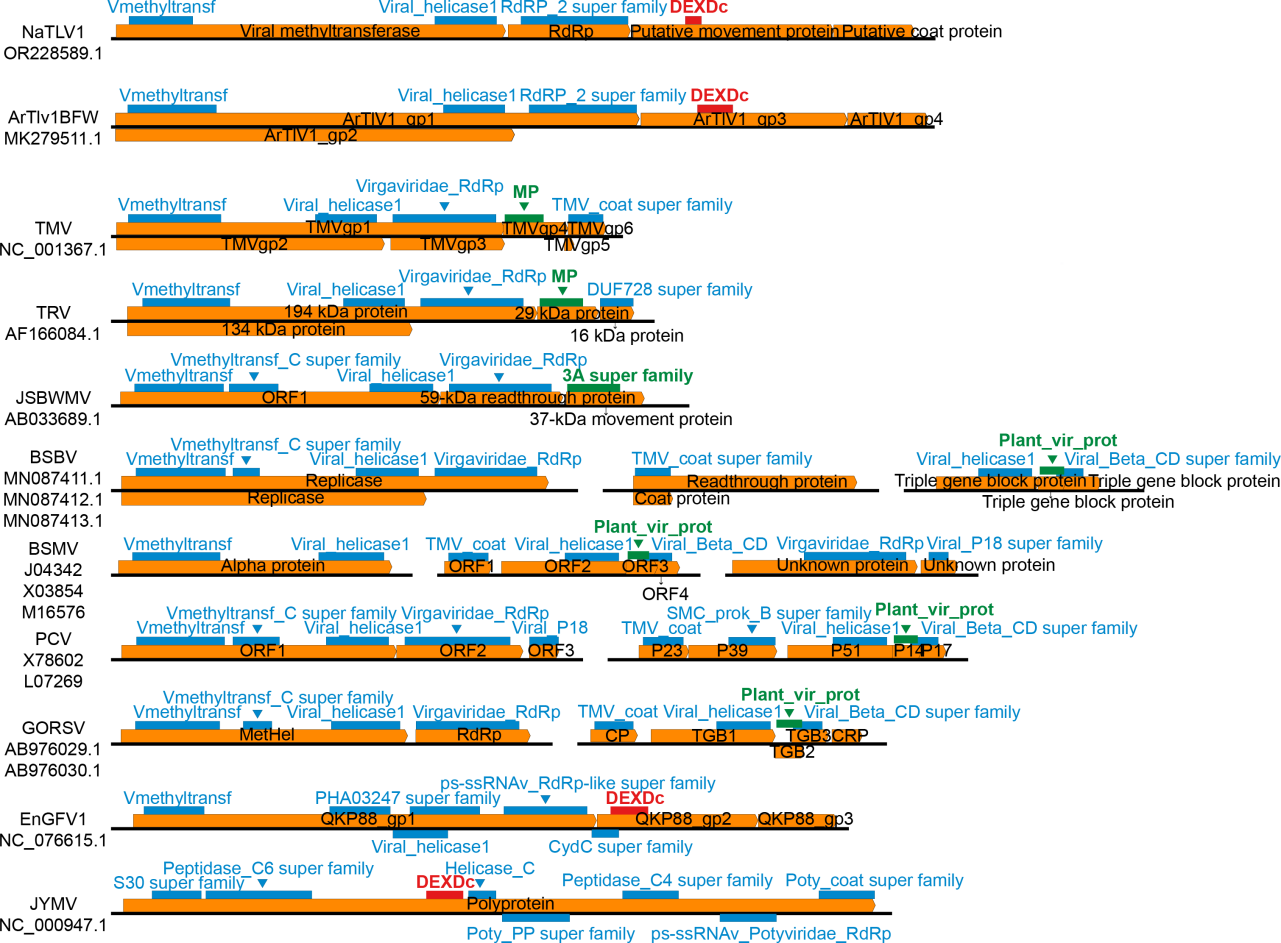
Comparison of the NaTLV1 genome structure with those of all representative strains of the *Virgaviridae* family and representative strains exhibiting homology with the NaTLV1 MP. The virus names and GenBank accession numbers are listed on the left. Black lines represent the whole viral genome, and orange boxes indicate open reading frames. Red rectangles represent DEXDc protein domains, green rectangles represent MP domains, and blue rectangles represent other protein domains. The names of the protein domains are labeled nearby in their corresponding colors. The names of the proteins are labeled in black font within the orange open reading frames.

To further investigate the differences between the putative MP of NaTLV1 and the MPs of other *Virgaviridae* members, we compared the genome structure, protein domains, and protein functions of NaTLV1, representative tobamo-like viruses, and representative members of the *Virgaviridae* family (Fig. 3; Table S6). The putative MPs of NaTLV1 and related tobamo-like viruses contain the DEXDc domain (which is involved in RNA unwinding), while the MPs of other *Virgaviridae* members contain the MP, 3A superfamily, or Plant_vir_prot domains (all of which are involved in viral movement). Additionally, the putative MP of NaTLV1 exhibited similarity to the MP of Entoleuca gammaflexivirus 1 (family *Gammaflexiviridae*, order *Tymovirales*, class *Alsuviricetes*) and the cylindrical inclusion protein (CI protein) of Japanese yam mosaic virus (family *Potyviridae*, order *Patatavirales*, class *Stelpaviricetes*) (Table S7). Furthermore, protein domain analysis revealed that the homologous regions among them contain the DEXDc domain.

**Fig 4:**
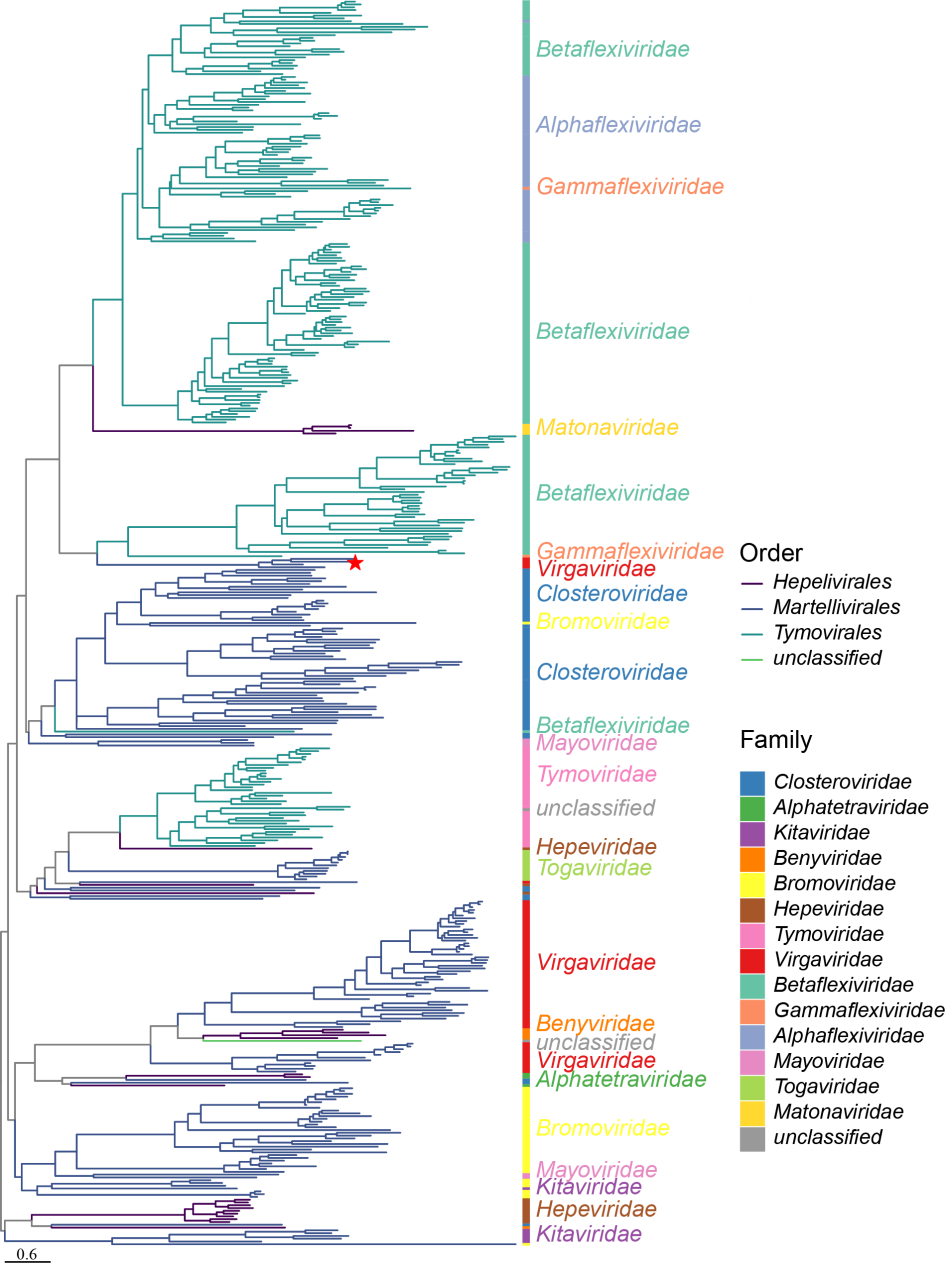
Phylogenetic analysis of CPs of major viruses in the *Alsuviricetes* class. The phylogenetic tree was estimated using a maximum-likelihood method based on CPs from various orders in the *Alsuviricetes* class, which are represented by branches of varying colors in the tree. The NaTLV1 identified in this study is marked with a red star. Family-level classifications are denoted by colored bars alongside the tree. Midpoint rooting was used. Branch scale bar = 0.6 substitutions per site.

Additionally, we constructed a phylogenetic tree based on the CPs of major members of the *Alsuviricetes* class to explore the morphological differences between NaTLV1 and the viruses of the *Virgaviridae* family (Fig. 4). We found that members of the *Martellivirales* and *Tymovirales* orders were distinctly separated, while members of the *Hepelivirales* order exhibited less clustering. Among viruses in the same branch, there were morphological similarities. For example, members of the *Tymoviridae* and *Togaviridae* families clustered together, and both exhibit a rounded contour morphology. Additionally, most members of the *Virgaviridae* family (excluding tobamo-like viruses) clustered with members of the *Benyviridae* family, and both exhibit a rigid, short, rod-like morphology. NaTLV1 clustered with several tobamo-like viruses and was adjacent to several members of the *Gammaflexiviridae* and *Betaflexiviridae* families. Viruses in the *Gammaflexiviridae* and *Betaflexiviridae* families are characterized by flexuous filaments, with diameters ranging from 10 to 15 nm (typically 12 nm–13 nm) and lengths ranging from 600 to 1,000 nm (https://talk.ictvonline.org/taxonomy/). Consequently, we hypothesized that the capsid morphology of NaTLV1 and related tobamo-like viruses would be more similar to that of members of the *Gammaflexiviridae* and *Betaflexiviridae* families (order *Tymovirales*) than members of the *Virgaviridae* family (order *Martellivirales*).

### Transmission of NaTLV1 and its effects on the host fungi

**Fig 5:**
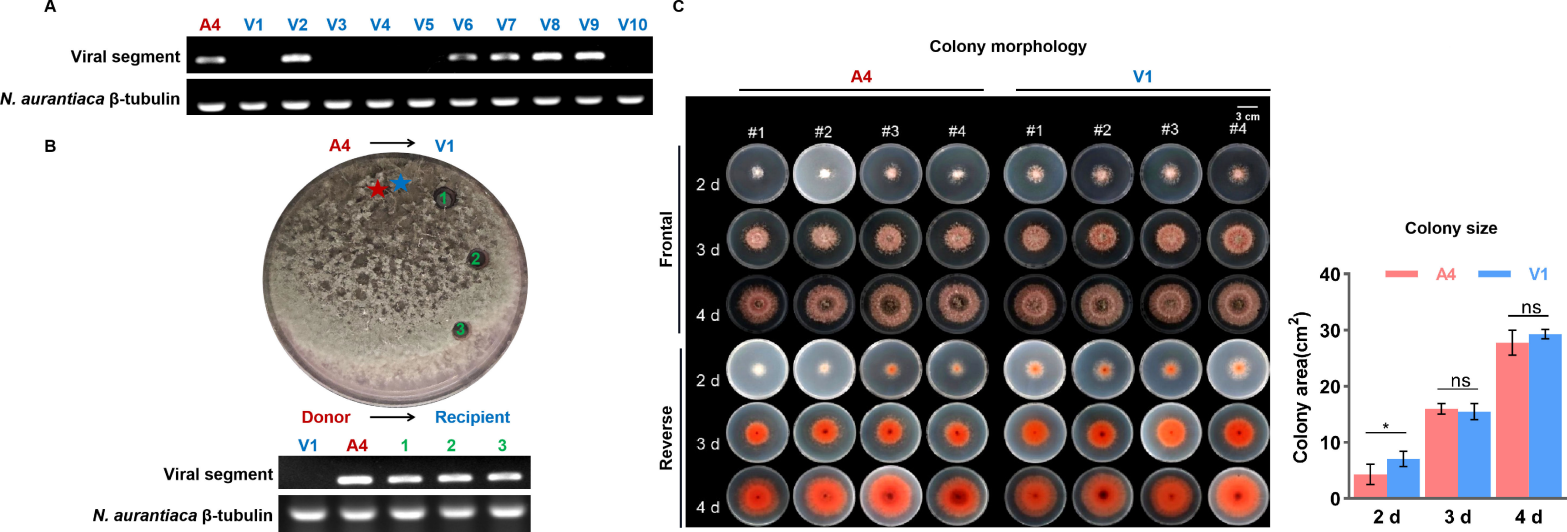
NaTLV1 transmission and its phenotypic effects on the host fungus. (**A**) NaTLV1 vertical transmission determined by RT-PCR-based detection of NaTLV1 genomic RNA using total RNA as the template. Owing to the difficulty of separating the NaTLV1 genomic segment on agarose gel, the genomic RNA was detected using RT-PCR. The β-tubulin gene of *N. aurantiaca* was used as the internal control. Ten subisolates (V1–V10) were selected from conidia harvested from young (7-day-old) mycelia of the NaTLV1-infected (**A4**) *N. aurantiaca* strain. (**B**) NaTLV1 horizontal transmission via hyphal anastomosis after co-culturing the NaTLV1-infected (**A4**) *N. aurantiaca* strain and NaTLV1-free (**V1**) *N. aurantiaca* strain on potato dextrose agar medium. Upper image: photograph of the plate after 10 days of co-culture. The NaTLV1 transmission from the donor to the recipient fungus is indicated by arrows, and numbers 1–3 indicate the positions from which the recipient mycelia were selected for subsequent culture. Lower image: RT-PCR-based detection of NaTLV1 accumulation in the recipient fungi (1–3). The β-tubulin gene of *N. aurantiaca* was used as the internal control. (**C**) Comparison of colony morphology and colony size (based on ImageJ; http://imagej.nih.gov/ij/) of the NaTLV1-infected (**A4**) *N. aurantiaca* strain and NaTLV1-free (**V1**) strain at 2–4 days. The mean values of four replicates are presented in a column chart, with error bars representing standard deviation. * indicates significant difference (*P* < 0.05) between the A4 and V1 strains based on the *t*-test.

Mycoviruses are commonly transmitted vertically via spores and horizontally through hyphal anastomosis (32); hence, we investigated the transmissions of NaTLV1 in *N. aurantiaca*. The rate of vertical transmission of NaTLV1 via fungal conidia was determined (using reverse transcription PCR [RT-PCR]) to be approximately 50% (Fig. 5A). The vegetatively compatible fungal strains were also shown to exhibit horizontal transmission, from NaTLV1-infected (A4) to NaTLV1-free (V1) *N. aurantiaca* (Fig. 5B).

The most known mycoviruses cause little or no obvious symptoms in their fungal hosts, such as Botrytis virus F and Botrytis virus X, to name a few (33, 34). However, some mycoviruses can reduce the virulence of their fungal hosts, typically *Cryphonectria hypovirus* 1, and they are valuable as biological control agents for fungi in plants (35). Therefore, we examined the effects of NaTLV1 on *N. aurantiaca* phenotype and virulence. The phenotypic characteristics of the NaTLV1-infected (A4) *N. aurantiaca* strain were compared to those of the NaTLV1-free (V1) strain, and the findings suggest that NaTLV1 infection did not markedly influence colony morphology or size (Fig. 5C). Moreover, light microscope observation and absorbance measurement (at 530 nm) showed that the conidia morphology and production also had no changes in the *N. aurantiaca* strain observed by NaTLV1 infection (Fig. S3). In addition, to evaluate the effect of NaTLV1 on *N. aurantiaca* virulence, we performed inoculation experiments. *N. aurantiaca* successfully infected and caused leaf spots on *N. benthamiana* (Fig. S4), but the NaTLV1 infection had no effects on the lesion areas of A4- and V1-inoculated *N. benthamiana* leaves for 7 days (Fig. S5). These results indicate that symptoms induced by NaTLV1 seem to have no effects on *N. aurantiaca* strain phenotype and virulence.

### Virus-like particles associated with NaTLV1

**Fig 6:**
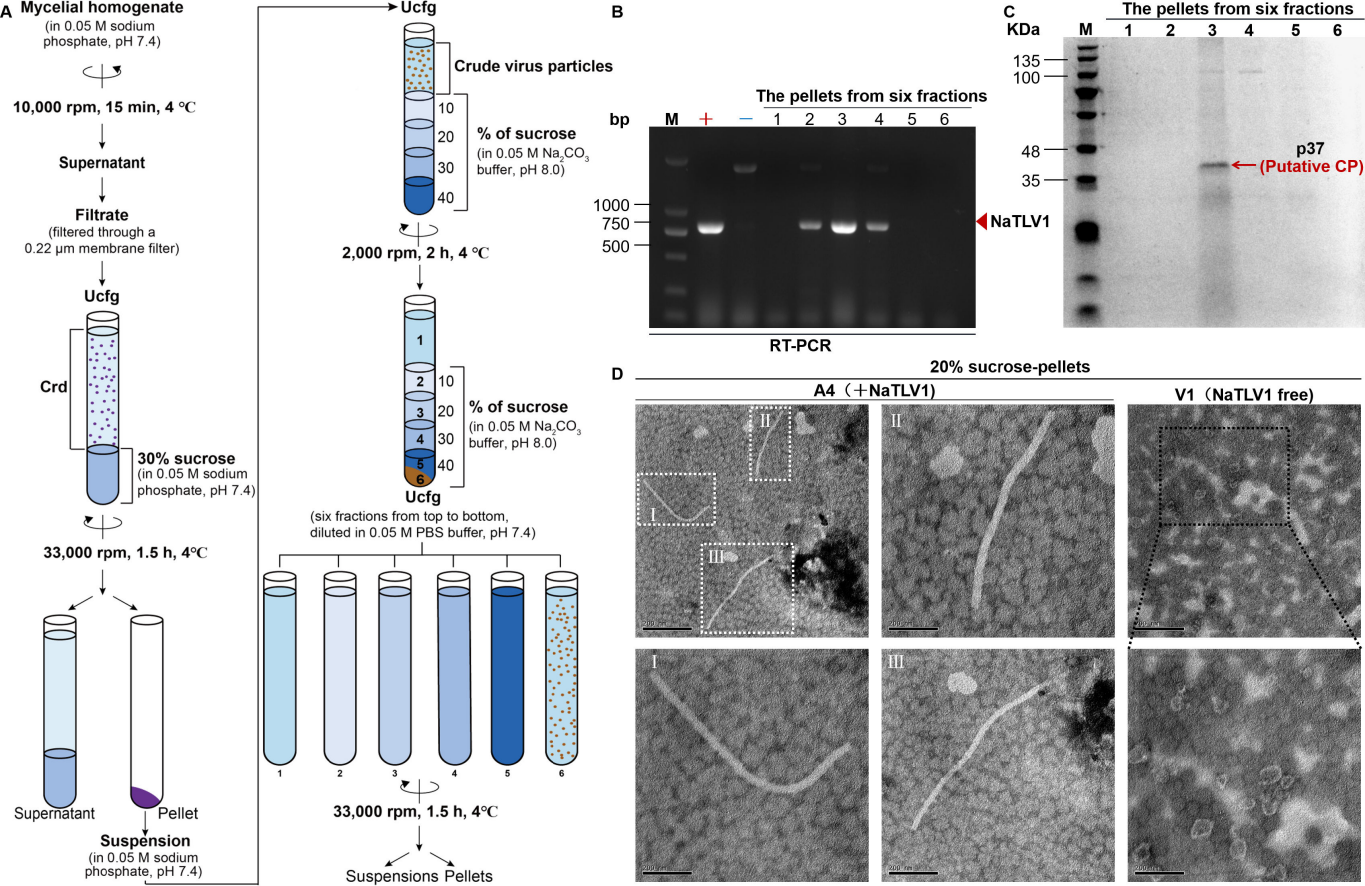
Purification of NaTLV1 virus particles from NaTLV1-infected (**A4**) *N. aurantiaca* strain. (**A**) Schematic representation of the fractionation of NaTLV1 virus particles by ultracentrifugation (Ucfg) involving a sucrose density gradient, which produced six fractions. The numbers 1 to 6 on the centrifuge tube indicate samples from the six fractions of supernatant, 10% to 40% sucrose layers, and the precipitate, respectively, from top to bottom of the centrifuge tube. Crd denotes the crude extract. (**B**) Electrophoretic profiles of RT-PCR products formed based on the nucleic acids in the pellets of six fractions from the NaTLV1-infected (**A4**) *N. aurantiaca* strain. “+” and “−” indicate strains A4 and V1, respectively. M, DNA size marker. (**C**) Sodium dodecyl sulfate polyacrylamide gel electrophoresis of proteins in the pellets of six fractions from the NaTLV1-infected (**A4**) *N. aurantiaca* strain. M, protein molecular weight marker. (**D**) Representative transmission electron microscopy images of the pellet of fraction 3 (20% sucrose fraction) purified from the A4 or V1 *N. aurantiaca* strain. Scale bar = 200 nm.

The BLASTp search and phylogenetic analysis revealed that the putative CPs of NaTLV1 and other unclassified tobamo-like viruses were most closely related to the CPs of certain viruses in the *Gammaflexiviridae* and *Betaflexiviridae* families. However, whether NaTLV1 and other unclassified tobamo-like viruses can form typical virus particles was unknown. Therefore, we attempted to purify NaTLV1 virus particles from the NaTLV1-infected (A4) *N. aurantiaca* strain using the experimental procedure shown in Fig. 6A. This procedure included ultracentrifugation involving a sucrose density gradient, which produced six fractions; these fractions were centrifuged to collect the pellets for further investigation.

Electrophoretic profiles of the RT-PCR products formed based on the nucleic acids in the pellets revealed the presence of the NaTLV1 RT-PCR fragment in fractions 2, 3, and 4 (10%, 20%, and 30% sucrose fractions, respectively) (Fig. 6B). Sodium dodecyl sulfate polyacrylamide gel electrophoresis (SDS-PAGE) of the proteins in the pellets revealed the presence of a dominant band with an estimated molecular mass of 37.0 kDa (p37) in the pellet of fraction 3 (20% sucrose fraction) (Fig. 6C).

To further examine p37, it was eluted from the gel and subjected to peptide mass fingerprinting. The results revealed that p37 generated 20 peptide fragments (Table S8). The sequences of the p37 fragments matched the deduced NaTLV1 CP sequence at aa 35–326 (accounting for 67.3% of the entire coverage). This shows that p37 was most likely NaTLV1 CP (encoded by NaTLV1 ORF4) (Table S8).

Additionally, the pellet of fraction 3 (20% sucrose fraction) was examined by transmission electron microscopy, which revealed filamentous virus-like particles of approximately 37 nm in width and 1,100 nm in length (Fig. 6D, left; Fig. S6). No filamentous virus-like particles were recovered from the NaTLV1-free (V1) *N. aurantiaca* strain (Fig. 6D, right).

Together, these results illustrate that NaTLV1 is encapsidated by CP, forming filamentous virus particles.

### NaTLV1 replicates, but cell-to-cell movement is not observed in the plant infection model

**Fig 7:**
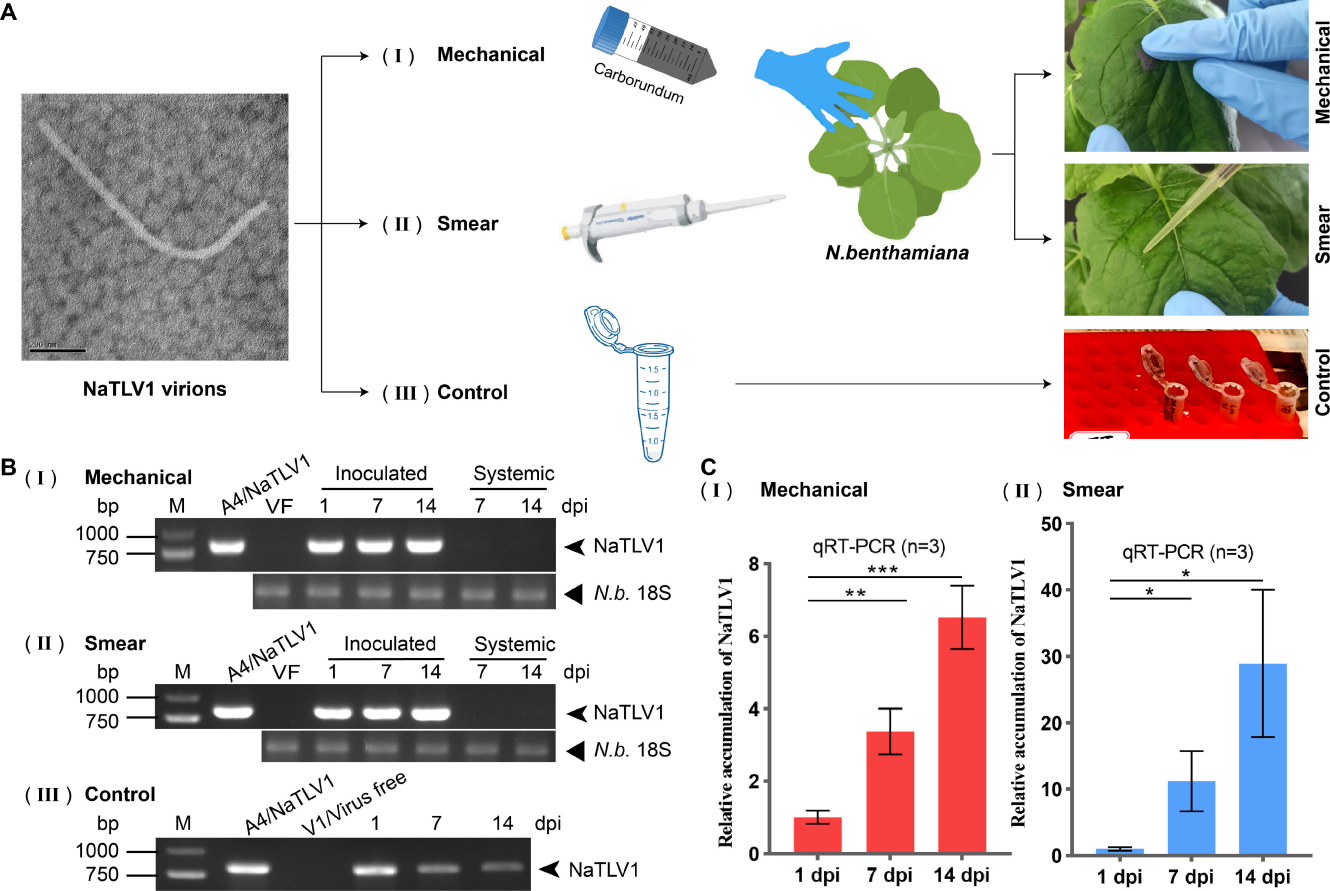
Infectivity of NaTLV1 virus particles in *N. benthamiana* plants. (**A**) Schematic representation of the experimental design and photographs demonstrating (**I**) mechanical friction inoculation with NaTLV1 virus particles, (II) smear inoculation with NaTLV1 virus particles, and (III) control (NaTLV1 virus particles in Eppendorf tubes in a plant incubator). Scale bar = 200 nm. In order to better represent NaTLV1 virions in Fig. 7A, we reused an image from Fig. 6D. (**B**) Agarose gel electrophoresis images of the RT-PCR-based detection of NaTLV1 accumulation in inoculated, upper non-inoculated *N. benthamiana* leaves and Eppendorf tubes as described in (**A**). VF, virus free; N.b., *N. benthamiana*. (**C**) qRT-PCR of NaTLV1 accumulation in inoculated *N.benthamiana* leaves (inoculated by mechanical friction inoculation or smear inoculation) as described in (**A**). *P < 0.05, **P < 0.01, ***P < 0.001 (Student’s *t*-test).

Bioinformatics analysis showed that the putative MPs of NaTLV1 and related tobamo-like viruses exhibited significant differences compared to the MPs of members of the *Virgaviridae* family. Thus, we further investigated NaTLV1 replication and cell-to-cell movement in *N. benthamiana* (Fig. 7A; Fig. S7A). The results showed that viral RNA was detected in the inoculated leaves from 1 to 14 days postinoculation (dpi); however, it was not present in the uninoculated upper leaves at 7 and 14 dpi (Fig. 7B). Meanwhile, the amount of NaTLV1 was lower at 14 dpi than at 1 dpi in the control group (Fig. 7B). In contrast, on inoculated *N. benthamiana* leaves, the NaTLV1 accumulation was higher at 14 dpi than at 1 dpi (Fig. 7B). To confirm NaTLV1 replication in inoculated leaves, NaTLV1 accumulation was assessed by quantitative RT-PCR (qRT-PCR). This confirmed that NaTLV1 accumulation gradually increased over time (1, 7, and 14 dpi) (Fig. 7C).

NaTLV1 accumulation in NaTLV1-inoculated and non-inoculated regions of a single *N. benthamiana* leaf at 14 dpi was detected by RT-PCR (Fig. S7A), which showed that NaTLV1 replication only occurred in the NaTLV1-inoculated regions (Fig. S7B). These results suggest that NaTLV1 does not undergo cell-to-cell movement in *N. benthamiana* leaves.

Together, these results demonstrate that NaTLV1 can replicate in *N. benthamiana* leaves; moreover, NaTLV1 cell-to-cell movement was not observed in *N. benthamiana* leaves, but it does not completely rule out cell-to-cell movement in the small sample size of non-inoculated tissue.

## DISCUSSION

The evolutionary relationships between fungal and plant viruses are highly complex, but there have been research breakthroughs. For instance, a study has provided for the first time evidence that two mycoviruses belonging to the *Partitiviridae* and *Totiviridae* families, which were respectively named Penicillium aurantiogriseum partitivirus 1 and Penicillium aurantiogriseum totivirus 1, can replicate in plant cells (36). Additionally, a recent study showed that a negative-strand RNA virus, named Valsa mali negative-strand RNA virus 1, related to members of the *Phenuiviridae* family, which naturally infects fungal and plant hosts, was found in the phytopathogenic fungus *Valsa mali*, which causes apple Valsa canker (37). Furthermore, certain plant viruses have been found to replicate in fungal hosts; for example, cucumber mosaic virus was found to naturally infect the fungus *Rhizoctonia solani* (9, 38–41). These studies provide evidence of cross-kingdom infections by fungal and plant viruses and give rise to further questions regarding the origins of plant and fungal viruses.

A group of tobamo-like viruses (recorded in GenBank) (Table S1) has recently been identified worldwide. These viruses generally possess a (+)ssRNA genome of 9 kb–13 kb that contains a short poly(A) tail and three or four ORFs (15, 18–22). These ORFs encode an RdRp domain, a putative MP, and a putative CP that are mainly homologous to corresponding proteins of plant-associated viruses in the *Tobamovirus* genus of *Virgaviridae* family, *Betaflexiviridae* family, and fungus-associated viruses in the *Gammaflexiviridae* family.

This study identified a novel tobamo-like virus, NaTLV1, from the phytopathogenic fungus *N. aurantiaca*. The whole genome of NaTLV1 consists of 10,301 nt and includes four ORFs and a poly(A) tail. ORF1 encodes Mtr and Hel proteins, and ORF2 contains an RdRp domain. The aa sequences of ORF1 and ORF2 are homologous to corresponding proteins in the *Tobamovirus* genus. Based on ICTV classification standards and phylogenetic analysis, NaTLV1 is most closely related to the *Virgaviridae* family, and it may form a distinct genus or family with other related tobamo-like fungal viruses.

NaTLV1 ORF3 encodes a putative MP that exhibited homology not only with the MPs of tobamo-like viruses and putative MPs of fungal viruses in the *Gammaflexiviridae* family, but also with CIs of plant viruses in the *Potyviridae* family. Additionally, we found that the putative MPs of tobamo-like viruses were notably longer than those of other genera in the *Virgaviridae* family. Protein domain analysis suggested that the putative MPs of tobamo-like viruses no longer function as MPs but exhibit domains such as the DEXDc domain, indicating helicase activity. This alteration may explain NaTLV1’s lack of cell-to-cell movement in *N. benthamiana*. Additionally, the conserved DEXDc domain of NaTLV1 MP was shared with the putative MPs of tobamo-like viruses and fungal viruses in the *Gammaflexiviridae* family, and with the CIs of plant viruses in the *Potyviridae* family. These findings suggest that NaTLV1 and some tobamo-like viruses represent a distinct group of fungal viruses, potentially evolving via reassortment and recombination of genomic segments from RNA viruses infecting various eukaryotes, particularly fungi and plants. This highlights novel connections between fungal and plant viruses at the gene level. Research indicates that viruses can co-opt eukaryotic DEAD/DEAH-box helicases to evade host immune detection and facilitate viral replication (42). This study showed that NaTLV1 can replicate in *N. benthamiana*, but there was degradation or loss of the movement function of the NaTLV1 MP (Fig. 7; Fig. S7). However, whether this genomic locus has acquired new gene functions remains uncertain. The origins of the DEAD-like helicase superfamily domain in plant and fungal viruses (whether co-opted from plants or fungi or other hosts), and the reasons for the convergent acquisition of this domain by different viruses, warrant further investigation.

The ORF4 of NaTLV1 encodes a putative CP, and sequence alignment using the NCBI BLASTn online tool (default parameters) showed that its nt sequence exhibited no homology with any known sequences. However, BLASTp results revealed aa sequence homology exclusively with tobamo-like viruses. Based on phylogenetic analysis (using representative CP aa sequences in the *Alsuviricetes* class), the cluster containing tobamo-like viruses, including NaTLV1, was closely associated with the *Gammaflexiviridae* and *Betaflexiviridae* families (Fig. 4). These families, which are fungi- and plant-infecting viruses, respectively, are characterized by a flexuous filament morphology (https://talk.ictvonline.org/taxonomy/). Our electron microscopy and phylogenetic analysis results suggest that the NaTLV1 morphology more closely resembles the flexuous rod morphology of viruses in the *Gammaflexiviridae* and *Betaflexiviridae* families, rather than the straight rod morphology (short virus particles, 32 nm–34 nm in length) typical of tobamoviruses (Fig. 6; Fig. S6).

In conclusion, the findings revealed that NaTLV1 and other tobamo-like viruses could be regarded as a new viral genus or family and are closely related to plant virus families (*Virgaviridae* and *Betaflexiviridae*) and a fungal virus family (*Gammaflexiviridae*), suggesting that they may have originated from a common ancestor or undergone recombination during evolution. In addition, the discovery of NaTLV1, a novel tobamo-like virus, supports the link between fungal and plant viruses, which also further expands our understanding of viral diversity, taxonomy, and evolution.

## MATERIALS AND METHODS

### Fungi, viruses, plants, and primers

The *N. aurantiaca* fungal strain (A4) infected with NaTLV1 was inadvertently isolated from a contaminated potato dextrose agar (PDA) plate, which had been gifted to our laboratory by another research team and subsequently stored at −80°C. Accidentally, the *N. aurantiaca* strain A4 with dsRNA accumulation was detected using CF11 cellulose (43). The NaTLV1-free (V1) isogenic subisolates were obtained by single-conidium isolation and confirmed to be NaTLV1-free by RT-PCR. All fungal strains were maintained on PDA or potato dextrose broth (PDB) at 28°C.

*N. benthamiana* was kindly provided by Prof. Yingtian Deng (Huazhong Agricultural University, Wuhan, China). *N. benthamiana* was grown in a plant growth chamber at 25 ± 2°C and a relative humidity of 70%–80%, with a 16/8 h (day/night) photoperiod.

The primers are shown in Table S4.

### NaTLV1 RNA extraction, sequencing, and bioinformatics analysis

The total RNA was extracted from the NaTLV1-infected (A4) *N. aurantiaca* strain using an EZNA Fungal RNA Kit (Omega, USA). Library preparation was conducted using an NEBNext Ultra II Directional RNA Library Prep Kit for Illumina, followed by sequencing on an Illumina NovaSeq 6000 (PE150) sequencing platform (Illumina, San Diego, CA, USA).

The raw reads were filtered using fastp v.0.20.0 (44), and the clean reads were assembled using SPAdes v.3.13.0 (45). The obtained contigs were compared against the non-redundant protein database by diamond blastx programme v.2.1.8.162 to identify tobamo-like virus (46). The abundance of the assembled sequence was estimated using a read mapping approach involving Bowtie2 v.2.2.5. The average number of reads per base of the assembled sequence was determined using Samtools v.1.10 (47).

Next, to further confirm the full-length genome of NaTLV1, 15 fragments with a 26- to 215-nt overlap, covering nearly the whole NaTLV1 genome, were amplified using RT-PCR followed by Sanger sequencing (Fig. 1; Table S4). Lastly, terminal fragments were obtained through 5´ and 3´ rapid amplification of cDNA ends using T4 RNA ligase oligonucleotide-mediated amplification with sequence-specific primers (Fig. 1; Table S4), as described previously (48).

The NaTLV1 RdRp nt sequence was aligned with the same fragments of various other members of the *Virgaviridae* family using MAFFT v.7.505 (https://mafft.cbrc.jp/alignment/software/). The NaTLV1 CP aa sequence was aligned with the same fragments of major members of the *Alsuviricetes* class using MAFFT v.7.505 (https://mafft.cbrc.jp/alignment/software/). Phylogenetic trees were constructed using the maximum-likelihood method in IQ-TREE v.2 with 1,000 bootstrap replicates. A domain analysis was conducted based on an NCBI Conserved Domain Search (https://www.ncbi.nlm.nih.gov/Structure/cdd/wrpsb.cgi).

### Horizontal and vertical transmission of NaTLV1 in *N. aurantiaca* and fungal strain characterization

Horizontal transmission of NaTLV1 from NaTLV1-infected (A4) to NaTLV1-free (V1) *N. aurantiaca* strains was assessed as previously described (49) with minor modifications. First, the same amount of NaTLV1-infected (A4) and NaTLV1-free (V1) *N. aurantiaca* strains were cocultured side-by-side on a PDA medium for 10 days. Following co-culture of the donor strain with the recipient strain, NaTLV1 was efficiently transmitted to the V1 strain through hyphal fusion. The mycelial agar plugs of the recipient strain were placed onto a fresh PDA plate and subjected to RT-PCR-based detection, as described in “RT-PCR confirmation of viral infection and RT-qPCR,” below.

The vertical transmission efficiency of NaTLV1 through the conidia of the NaTLV1-infected (A4) *N. aurantiaca* strain was assessed using single-conidium isolation (50), in conjunction with RT-PCR-based detection of the NaTLV1 genomic segment in subisolates. In brief, conidia produced on 7-day-old mycelia of the *N. aurantiaca* isolate A4 were suspended in sterilized water and spread onto PDA medium to obtain single conidial subisolates. The determination of whether A4 single conidial subisolates were positive or negative for NaTLV1 was conducted via RT-PCR, as outlined in “RT-PCR confirmation of viral infection and RT-qPCR,” below.

The phenotype and virulence of strains A4 and V1 were assessed. Briefly, mycelial plugs were cut from the actively growing colony margins of each strain and placed in the middle of a PDA Petri dish, and fungal morphology was observed. The growth rate was calculated daily by measuring the colony areas for 4 days. The absorbance at 530 nm was measured for each conidial suspension obtained from the *N. aurantiaca* strains to evaluate conidial production (51, 52). To assess virulence, *N. benthamiana* leaves were observed after mycelial plugs were inoculated on the leaves as described in the next section. The lesion areas of A4- and V1-inoculated *N. benthamiana* leaves were measured for 7 days to compare their virulence (37).

### Virus particles and fungal inoculation

*N. benthamiana* plants were inoculated with virus particles as previously described (9) with minor modifications. For mechanical friction inoculation with virus particles, the virus particles extracted from the NaTLV1-infected (A4) fungal mycelia were rubbed onto carborundum (silicon carbide)-dusted *N. benthamiana* leaves. For smear inoculation with virus particles, a pipette gun was used to smear the virus particles (equal to the amount used for mechanical friction inoculation) onto *N. benthamiana* leaves. For inoculation with the A4 and V1 *N. aurantiaca* strains, lower *N. benthamiana* leaves were wounded using sterilized toothpicks, and mycelia-containing gel plugs (around 0.5 × 0.5 cm) from the edge of a 4-day-old colony were placed on the wounded area. The inoculated plants were cultured for about 5 or 7 days.

### RT-PCR confirmation of viral infection and RT-qPCR

RT-PCR was performed to confirm the existence of viral genome RNA in the mycelia, as outlined in “Virus particle ssRNA and protein analysis,” below. Total RNA was extracted from the *N. benthamiana* leaves using TRIzol (Invitrogen) or from *N. aurantiaca* mycelia by phenol/chloroform extraction. The RT-qPCR templates were synthesized using Hifair II 1st Strand cDNA Synthesis superMix (Yeasen, Shanghai, China). Next, the qRT-PCR was performed using 2×Q3 SYBR qPCR Master Mix (Universal) on a Real-Time PCR Detection System (QuantStudio 3). *N. benthamiana* EF1α RNA was employed as an internal control for quantitative RT-PCR, as described previously (53). Experiments were performed in three independent replicates, and each sample was analyzed in triplicate. The free statistical software Jamovi was used to run the analysis (version 1.6.15) (54).

### Virus particle purification and visualization

Virus particle purification was conducted as previously described (55), with slight modifications. Briefly, the fungal mycelia were cultured in PDB for 4 days at 28°C, ground in liquid nitrogen using a pestle and mortar, homogenized in three volumes (vol/wt) of 0.05 M sodium phosphate (pH 7.4), centrifuged (10,000 rpm and 4°C for 10 min), and then filtered through a 0.22 µm membrane filter to collect the filtrate. The filtrate was overlaid onto a 30% (wt/vol) sucrose cushion at a volume ratio of 2:1 and ultracentrifuged (33,000 rpm and 4°C for 1.5 h) using an Optima XPN-100 Ultracentrifuge (Beckman, USA). The pellet was suspended in 0.05 M phosphate-buffered saline (PBS), applied on top of a 10%–40% sucrose density gradient in 0.05 M Na_2_CO_3_ buffer (pH 8.0), and centrifuged (20,000 rpm and 4°C for 2 h) using a SW41Ti rotor (Beckman). Next, the gradient was separated into six fractions (from top to bottom), diluted in 0.05 M PBS buffer, and centrifuged (33,000 rpm and 4°C for 1.5 h). Each pellet was suspended in 100 µL of 0.05 M PBS. Purified virus particle preparations were examined by transmission electron microscopy, as previously described (49). The length and width of the virus particles were measured using ImageJ.

### Virus particle ssRNA and protein analysis

First, the ssRNA in each of the abovementioned six fractions was purified using phenol:chloroform:isoamyl alcohol extraction as previously described (24). Briefly, 50 µL sucrose suspension was collected from each fraction and treated with phenol:chloroform:isoamyl alcohol (25:24:1; pH 5.2) to remove the viral proteins. The nucleic acids were precipitated with ethanol and dissolved in diethylpyrocarbonate-treated water. To verify the presence of mycoviruses, cDNA was synthesized from the precipitated nucleic acids using Moloney murine leukemia virus reverse transcriptase (TaKaRa, Dalian, China), amplified using Taq DNA polymerase (TaKaRa, Dalian, China), and analyzed by agarose gel electrophoresis.

Second, the proteins in each of the six fractions were separated by 12% SDS-PAGE in a 25 mM Tris-glycine and 0.1% SDS buffer. The gels were stained with Coomassie brilliant blue R-250 (Bio-Safe CBB; Bio-Rad, USA). The protein bands were individually excised and subjected to peptide mass fingerprinting analysis (56) by APTBIO (Shanghai) Co., Ltd. (China).

## Data Availability

The data reported in this paper were deposited in the NCBI GenBank database under accession number OR228589.1.

## SUPPLEMENTAL MATERIAL

Supplemental material jvi.02102-24-s0001.docx Tables S1 to S8; Fig. S1 to S7.

ASM does not own the copyrights to Supplemental Material that may be linked to, or accessed through, an article. The authors have granted ASM a non-exclusive, world-wide license to publish the Supplemental Material files. Please contact the corresponding author directly for reuse.
