# Supplementary material for: A novel tobamo-like mycovirus with filamentous particles replicates in plant cells
Source: J Virol. 2025 Mar 31;99(5):e02102-24. doi: 10.1128/jvi.02102-24 (PMC12090730; doi:10.1128/jvi.02102-24)
Supplement: Supplemental material — Tables S1 to S8; Fig. S1 to S7. [file jvi.02102-24-s0001.docx]

**Table S1.** The information of Fungus associated Tobamo-like viruses in the GenBank database.

| **Name** | **Sequence integrity** | **GenBank accession no.** | **Length (bp)** | **Collection date** | **Published** | **Country** |
| --- | --- | --- | --- | --- | --- | --- |
| Auricularia heimuer mycovirgavirus 1 | complete | MN928963.1 | 9934 | 2019 | no | China |
| Heterobasidion tobamo-like virus 1 | complete | ON014539.1 | 12586 | 2019 | no | Czech Republic |
| Wheat associated tobamo-like virus | complete | OK573480.1 | 10285 | 2019 | yes | USA |
| Erysiphe necator associated tobamo-like virus 2 | partial | MN630179.1 | 3408 | 2018 | no | Spain |
| Erysiphe necator associated tobamo-like virus 1 | complete | MN627474.1 | 11430 | 2018 | no | Spain |
| Plasmopara viticola lesion associated tobamo-like virus 1 | complete | MN565665.1 | 11400 | 2018 | yes | Spain |
| Acidomyces richmondensis tobamo-like virus 1 | complete | MK279511.1 | 10291 | 2017 | yes | USA |
| Botryosphaeria dothidea tobamo-like virus | partial | MK189194.1 | 3687 | 2016 | no | China |
| Leucocoprinus tobamovirus A | partial | MK231117.1 | 9481 | 2016 | no | Panama |
| Podosphaera prunicola tobamo-like virus | complete | KY420046.1 | 11449 | 2015 | yes | USA |
| Armillaria borealis mycovirgavirus 1 | complete | MW423800.1 | 11258 | 2014 | yes | Russia |
| Macrophomina phaseolina tobamo-like virus 2 | partial | MT062438.1 | 3671 | 2011/2015 | yes | China |
| Macrophomina phaseolina tobamo-like virus-A | partial | MT062440.1 | 3417 | 2011/2015 | yes | China |
| Macrophomina phaseolina tobamo-like virus-B | partial | MT062441.1 | 3155 | 2011/2015 | yes | China |
| Macrophomina phaseolina tobamo-like virus-C | partial | MT062442.1 | 4677 | 2011/2015 | yes | China |
| Macrophomina phaseolina tobamo-like virus-D | partial | MT062443.1 | 2381 | 2011/2015 | yes | China |
| Macrophomina phaseolina tobamo-like virus 1a-A | partial | MT062439.1 | 2073 | 2011/2015 | yes | China |
| **Macrophomina phaseolina tobamo-like virus 1a** | partial | KP900897.2 | 7499 | 2013 | yes | USA |
| Macrophomina phaseolina tobamo-like virus | complete | KF537660.1 | 9524 | 2003 | yes | unknown |

| **Name** | **GenBank accession no.** | **Sequence integrity** | **Query coverage (%)** | **Identity (%)** | **E value** |
| --- | --- | --- | --- | --- | --- |
| Auricularia heimuer mycovirgavirus 1 | MN928963.1 | complete | - | - | - |
| Heterobasidion tobamo-like virus 1 | ON014539.1 | complete | - | - | - |
| Wheat associated tobamo-like virus | OK573480.1 | complete | 2 | 67.19 | 2.00E-10 |
| Erysiphe necator associated tobamo-like virus 2 | MN630179.1 | partial | - | - | - |
| Erysiphe necator associated tobamo-like virus 1 | MN627474.1 | complete | 3 | 65.54 | 6.00E-05 |
| Plasmopara viticola lesion associated tobamo-like virus 1 | MN565665.1 | complete | 3 | 65.54 | 6.00E-05 |
| Acidomyces richmondensis tobamo-like virus 1 | MK279511.1 | complete | 6 | 67.21 | 6.00E-11 |
| Botryosphaeria dothidea tobamo-like virus | MK189194.1 | partial | - | - | - |
| Leucocoprinus tobamovirus A | MK231117.1 | partial | - | - | - |
| Podosphaera prunicola tobamo-like virus | KY420046.1 | complete | 4 | 69.43 | 5.00E-12 |
| Armillaria borealis mycovirgavirus 1 | MW423800.1 | complete | - | - | - |
| Macrophomina phaseolina tobamo-like virus 2 | MT062438.1 | partial | - | - | - |
| Macrophomina phaseolina tobamo-like virus-A | MT062440.1 | partial | - | - | - |
| Macrophomina phaseolina tobamo-like virus-B | MT062441.1 | partial | - | - | - |
| Macrophomina phaseolina tobamo-like virus-C | MT062442.1 | partial | - | - | - |
| Macrophomina phaseolina tobamo-like virus-D | MT062443.1 | partial | - | - | - |
| Macrophomina phaseolina tobamo-like virus 1a-A | MT062439.1 | partial | 1 | 68.29 | 0.002 |
| **Macrophomina phaseolina tobamo-like virus 1a** | KP900897.2 | partial | 2 | 71.86 | 2.00E-18 |
| Macrophomina phaseolina tobamo-like virus | KF537660.1 | complete | 1 | 70.67 | 0.008 |

**Table S2.** Results of BLASTn searches with the nucleotide between the longest contig obtained by Illumina and the other fungus-associated tobamo-like viruses in the GenBank database.

**Table S3.** Results of BLASTp searches with the proteins between the longest contig obtained by Illumina and the other fungus-associated tobamo-like viruses in the GenBank database.

|  | | **Provisional top 3 results (lowest E value)** | | | | |
| --- | --- | --- | --- | --- | --- | --- |
| **Query** | **Conserved domain** | **Description** | **Query coverage (%)** | **Identity**  **(%)** | **E value** | **GenBank**  **accession no.** |
| ORF1 | methyltransferase; RNA helicase | Replicase  (Acidomyces richmondensis tobamo-like virus 1) | 77 | 33.08 | 1e-122 | AZT88674.1 |
|  |  | Replicase  (Macrophomina phaseolina tobamo-like virus) | 77 | 33.73 | 2e116 | AII21815.1 |
|  |  | Replicase  (Podosphaera prunicola tobamo-like virus) | 82 | 29.70 | 2e-103 | ATS94406.1 |
| ORF2 | RNA-dependent RNA Polymerase | RNA-dependent RNA polymerase  (Macrophomina phaseolina tobamo-like virus-C) | 74 | 33.29 | 4e-125 | QOE55602.1 |
|  |  | RNA-dependent RNA polymerase (Acidomyces richmondensis tobamo-like virus 1) | 77 | 33.08 | 1e-120 | AZT88673.1 |
|  |  | RNA-dependent RNA polymerase(Macrophomina phaseolina tobamo-like virus) | 77 | 33.73 | 1e-114 | AII21816.1 |
| ORF3 | DEAD-like helicase superfamily | Putative movement protein (Acidomyces richmondensis tobamo-like virus 1) | 100 | 49.07 | 0 | AZT88675.1 |
|  |  | movement protein  (Podosphaera prunicola tobamo-like virus) | 88 | 48.27 | 0 | ATS94408.1 |
|  |  | Putative polyprotein  (Erysiphe necator associated tobamo-like virus 1) | 100 | 45.26 | 0 | QKN22703.1 |
| ORF4 | None | Putative coat protein  (Acidomyces richmondensis tobamo-like virus 1) | 97 | 44.18 | 3e-99 | AZT88676.1 |
|  |  | coat protein  (Erysiphe necator associated tobamo-like virus 1) | 98 | 42.51 | 1e-86 | QKN22702.1 |
|  |  | coat protein (Podosphaera prunicola tobamo-like virus) | 99 | 40.18 | 1e-79 | ATS94409.1 |

**Table S4.** The primers were used in this study.

| **Name** | **Sequence (5**' **to 3**'**)** | **Oligos position in the NaTLV1 Genome (5**' **to 3**'**)** | **Size of PCR product** | **Remarks** |
| --- | --- | --- | --- | --- |
| REV-anchor | GCATTGCATCATGATCGATCGAATTCTTTA  GTGAGGGTTAATTGCC-(NH_2_)  (5' end phosphorylated oligonucleotide) | - | - | 5' and 3' RACE**^a^** |
| oligoREV-RT | GGCAATTAACCCTCACTAAAG | - | - |  |
| REV-PCR | TCACTAAAGAATTCGATCGATC | - | - |  |
| Virus-mo-5-1 | GTATCGGAACTGAACTGAGCCTGC | 1-191 | 191 bp |  |
| Virus-mo-5-2 | CTATCGTCAGCTGACAGTGCCAC | 1-251 | 251 bp |  |
| Virus-mo-3-2 | CAGTGACAGCGGTTATAGCGATTG | 9881-10301 | 251 bp |  |
| Virus-mo-3-4 | GATCGTGTGAGCGAAAGATACGGG | 9909-10251 | 421 bp |  |
| Virus-mo-3-5 | GAACGAGGACAAGAGATCGCCTG | 9992-10270 | 279 bp |  |
| Virus-F1 | GTTGCAGGCTCAGTTCAGTTCCG | 165-862 | 698 bp | Internal amplification**^a^** |
| Virus-R1 | CTTCCACGTCCTCAGTGATCATGC |  |  |  |
| Virus-F2 | GTGCGTGGCTACTCCTGTCATTG | 808-1545 | 738 bp |  |
| Virus-R2 | GTTCAACAAGCCGGACATGCTC |  |  |  |
| Virus-F3 | CGTAGAGACCTGCGCAAGAAGAG | 1468-2257 | 790 bp |  |
| Virus-R3 | CGGCTGACACAAGAGCGTCTAC |  |  |  |
| Virus-F4 | GAGTGATTCCGTTACGCCGAGTG | 2076-2822 | 747 bp |  |
| Virus-R4 | GGCTCGTCTTCAGTCGAGAGAC |  |  |  |
| Virus-F5 | GTGATTCGTCTAACACCAGGTCGC | 2696-3472 | 777 bp |  |
| Virus-R5 | CATCGACAACAGCCTTCACCTC |  |  |  |
| Virus-F6 | GGTGAAGGACTTGCGGACAGATC | 3414-4167 | 754 bp |  |
| Virus-R6 | CTTGAACTTCTGCTGGAGCTCTTC |  |  |  |
| Virus-F7 | CGACATAGTGGCAGTTCAGTCAACC | 4110-4971 | 862 bp |  |
| Virus-R7 | GCTTGACATGTCGCCAGTTAGAG |  |  |  |
| Virus-F8 | GACTACGTGACGATAGAGCAGACC | 4807-5635 | 829 bp |  |
| Virus-R8 | CCGATCTCGTTATCGGTCCGAAG |  |  |  |
| Virus-F9 | CACAGAATACTACGACGTTGTCCGC | 5420-6166 | 747 bp |  |
| Virus-R9 | CAATCTGACTGTACAGGGTCTTGGC |  |  |  |
| Virus-F10 | GCGTCTGAAGTCGGAGGAAGTC | 6006-6771 | 766 bp |  |
| Virus-R10 | CGCCAACCTCTCCACTGAAGTTC |  |  |  |
| Virus-F11 | GCCAGTTGATTCGGCTGAGTTC | 6696-7420 | 725 bp |  |
| Virus-R11 | CGGACGGTGCAATGAACGATAC |  |  |  |
| Virus-F12 | CTTGCTCGCAGAATAGGTGCAC | 7363-8132 | 770 bp |  |
| Virus-R12 | CTTGAGGATGGTGGATCCGTATCG |  |  |  |
| Virus-F13 | CGGTCAGGTATGACGGTAAGGTG | 7985-8747 | 763 bp |  |
| Virus-R13 | GTGACACCGTTTGGTTGTGTAGTC |  |  |  |
| Virus-F14 | GAAGCAAGTGTTCTCTGGCAGTG | 8610-9351 | 742 bp |  |
| Virus-R14 | CCTTCTTCGCTTCGGTCTCAGTAC |  |  |  |
| Virus-F15 | GTTGCCGAATCGGAAGTGGAAG | 9294-10014 | 721 bp |  |
| Virus-R15 | CAGGCGATCTCTTGTCCTCGTTC |  |  |  |
| ITS1 | TCCGTAGGTGAACCTGCGG | - | 189 bp | ITS-PCR |
| ITS4 | TCCTCCGCTTATTGATATGC | - |  |  |
| Bt2a | GGTAACCAAATCGGTGCTGCTTTC | - | 426 bp | TUB-PCR |
| Bt2b | ACCCTCAGTGTAGTGACCCTTGGC | - |  |  |
| Nb-18SF | GCAAGACCGAAACTCAAAGG | - | 216 bp | Nb18S-PCR |
| Nb-18SR | TGTTCATATGTCAAGGGCTGG | - |  |  |
| NaTLV1-CPF1 | GACCTGGAAGTTGCCGAATCG | 9285-9453 | 169 bp | qRT-PCR |
| NaTLV1-CPR1 | GCTCTTCGGAGAAGCTCGTT |  |  |  |
| NbEF1 α-F | AGCTTTACCTCCCAAGTCATC | - | 135 bp |  |
| NbEF1 α-R | AGAACGCCTGTCAATCTTGG | - |  |  |
| Random Primer 6 | 5'-P-d(NNNNNN)-3' | - | - | RT-PCR |

**^a^**Position of the primers on the genome of NaTLV1 are shown in **Fig. 1**.

**Table S5.** Results of BLASTp searches with the ORFs of NaTLV1.

|  | | **Provisional top 3 results (lowest E value)** | | | | |
| --- | --- | --- | --- | --- | --- | --- |
| **Query** | **Conserved domain** | **Description** | **Query coverage (%)** | **Identity**  **(%)** | **E value** | **GenBank**  **accession no.** |
| Fusion protein (ORF1 and ORF2) | methyltransferase; RNA helicase; RNA-dependent RNA Polymerase | Putative replicase readthrough protein  (endogenous Eutypa lata tobamo-like virus) | 79 | 43.49 | 0 | EMR61648.1 |
|  |  | RNA-dependent RNA polymerase  (Macrophomina phaseolina tobamo-like virus) | 82 | 43.26 | 0 | YP_009109559.1 |
|  |  | RNA-dependent RNA polymerase(Acidomyces richmondensis tobamo-like virus 1) | 83 | 44.78 | 0 | AZT88673.1 |
| ORF3 | DEAD-like helicase superfamily | Putative movement protein (Acidomyces richmondensis tobamo-like virus 1) | 99 | 49.07 | 0 | AZT88675.1 |
|  |  | Putative movement protein  (Macrophomina phaseolina tobamo-like virus 1a) | 99 | 46.64 | 0 | ANE37542.1 |
|  |  | Putative polyprotein  (endogenous Eutypa lata tobamo-like virus) | 79 | 54.03 | 0 | EMR61651.1 |
| ORF4 | None | Putative coat protein  (Mycosphaerella tobamovirus B) | 95 | 45.26 | 5e-94 | QED42913.1 |
|  |  | Putative coat protein  (Acidomyces richmondensis tobamo-like virus 1) | 97 | 44.18 | 3e-91 | AZT88676.1 |
|  |  | Hypothetical protein K491DRAFT_718881 [Lophiostoma macrostomum CBS 122681] | 90 | 43.93 | 4e-83 | KAF2652524.1 |

**Table S6.** Comparison of the Structure and function of the putative movement protein in representative strains of the family *Virgaviridae*.

| **Genus** | **Name of Representative Strain** | **Accession No.** | **Length (aa)** | **Host** | **Protein** | **Domain** | **Function** |
| --- | --- | --- | --- | --- | --- | --- | --- |
| - | Nigrospora aurantiaca tobamo-like virus 1 | WOC33730.1 | 834 | *Nigrospora aurantiaca*/Fungi | putative movement protein | DEXDc | RNA Unwinding |
| - | Acidomyces richmondensis tobamo-like virus 1 | AZT88675.1 | 859 | *Acidomyces richmondensis*/Fungi | putative movement protein | DEXDc | RNA Unwinding |
| *Tobamovirus* | Tobacco mosaic virus | NC_001367.1 | 269 | tobacco/Plant | movement protein | MP | involved in virus transport |
| *Tobravirus* | Tobacco rattle virus | AAD48028.1 | 252 | tobacco/Plant | 29 kDa protein | MP | involved in virus transport |
| *Furovirus* | Japanese soil-borne wheat mosaic virus | BAA94798.1 | 324 | wheat/Plant | 37-kDa movement protein | 3A super family | involved in virus transport |
| *Pomovirus* | Beet soil-borne virus | QEY10202.1 | 427 | Beet/Plant | triple gene block protein | Viral (Superfamily 1) RNA helicase | viral RNA replication |
|  |  | QEY10203.1 | 116 |  | triple gene block protein | Plant_vir_prot | presumably cell to cell movement |
|  |  | QEY10204.1 | 190 |  | triple gene block protein | Viral Beta C/D like family | conserved region found in the Beta C and Beta D transcripts |
| *Hordeivirus* | Barley stripe mosaic virus | AAA79161.1 | 512 | Barley stripe/Plant | beta-b protein | Viral_helicase1 | viral RNA replication |
|  |  | AAA79162.1 | 131 |  | beta-c protein | Plant_vir_prot | presumably cell to cell movement |
|  |  | AAA79163.1 | 155 |  | beta-d protein | Viral Beta C/D like family | conserved region found in the Beta C and Beta D transcripts |
| *Pecluvirus* | Peanut clump virus | AAA17438.1 | 446 | Peanut/Plant | P51 | Viral_helicase1 | viral RNA replication |
|  |  | AAA17439.1 | 122 |  | P14 | Plant_vir_prot | presumably cell to cell movement |
|  |  | AAA17440.1 | 153 |  | P17 | Viral Beta C/D like family | conserved region found in the Beta C and Beta D transcripts |
| *Goravirus* | Gentian ovary ring-spot virus | BAP18645.1 | 518 | Gentiana triflora/Plant | putative triple gene block 1 | Viral_helicase1 | viral RNA replication |
|  |  | BAP18646.1 | 120 |  | putative triple gene block 2 | Plant_vir_prot | presumably cell to cell movement |
|  |  | BAP18647.1 | 153 |  | putative triple gene block 3 | Viral Beta C/D like family | conserved region found in the Beta C and Beta D transcripts |

**Table S7.** Domain identification of homologous region of representative viral strains with amino acid sequence homology to the putative movement protein of NaTLV1.

| **Classification (class/order/family)** | **Name of  Representative Strain** | **Coverage/identity with purative  movement protein of NaTLV1** | **Accession No. of  homologous region** | **Length (aa)** | **Host** | **Protein** | **Domain** | **Function** |
| --- | --- | --- | --- | --- | --- | --- | --- | --- |
| *Alsuviricetes/Martellivirales/Virgaviridae* | Nigrospora aurantiaca tobamo-like virus 1 | —— | WOC33730.1 | 834 | *Nigrospora aurantiaca*/Fungi | putative movement protein | DEXDc | RNA Unwinding |
| *Alsuviricetes/Martellivirales/Virgaviridae* | Acidomyces richmondensis tobamo-like virus 1 | 99%/49.07% | AZT88675.1 | 859 | *Acidomyces richmondensis*/Fungi | putative movement protein | DEXDc | RNA Unwinding |
| *Alsuviricetes/Tymovirales/Gammaflexiviridae* | Entoleuca gammaflexivirus 1 | 74%/26.86% | YP_010799288.1 | 670 | *Entoleuca*/Fungi | movement protein | DEXDc | RNA Unwinding |
| *Stelpaviricetes/ Patatavirales/Potyviridae* | Japanese yam mosaic virus | 37%/24.77% | NP_734227.1 | 644 | Japanese yam/Plant | CI protein | DEXDc | RNA Unwinding |

**Table S8.** Summary of the peptide mass fingerprinting analysis of p37 encoded by ORF4 of NaTLV1 ssRNA.

| **Amino acid position** | **Mass*** | **Amino acid sequence** | **Score**** |
| --- | --- | --- | --- |
| 35-52 | 2043.9861 | TRDLDGVPTDITDADWVR | 80.238 |
| 37-52 | 1786.8374 | DLDGVPTDITDADWVR | 134.9 |
| 53-83 | 2855.4236 | MNQAGGAGPSGGGATGVVAPTPTIFDSLGIR | 120.13 |
| 84-106 | 2483.068 | MQDLEVAESEVEAGASCTETEAK | 111.67 |
| 107-119 | 1407.7762 | KVFEAAEALVGFK | 34.692 |
| 108-119 | 1279.6812 | VFEAAEALVGFK | 99.653 |
| 123-135 | 1634.8358 | AHFLINFIDWGFR | 145.89 |
| 136-149 | 1540.7046 | TSFSEELTEAGGWK | 139.86 |
| 165-173 | 950.56615 | VSVLHAAVR | 80.229 |
| 174-183 | 1085.4738 | DHLGESDTGR | 191.85 |
| 214-221 | 878.39162 | VGTDMSNR | 107.19 |
| 222-244 | 2648.3383 | LGVAPAFFLCCTSIFESIKPYTK | 55.173 |
| 245-254 | 1247.5571 | WSDDELAAWR | 157.68 |
| 255-262 | 709.38712 | AHNAAVK | 113.08 |
| 270-283 | 1651.81 | RPEDLRPDPEMAAR | 36.392 |
| 284-295 | 1404.5582 | FSDSGYSDWADR | 129.16 |
| 300-311 | 1236.5523 | YGHSGPSGQYGK | 202.96 |
| 318-326 | 1190.5615 | RMWGNEDKR | 60.436 |
| 319-325 | 878.35925 | MWGNEDK | 64.803 |
| 319-326 | 1034.4604 | MWGNEDKR | 40.002 |
| Note：Percentage of the sequence that is covered by the identified peptides of the best protein sequence contained in the group is 67.3％. * and **, monoisotopic mass of the peptide and andromeda score for the best associated MS/MS spectrum, respectively. | | | |


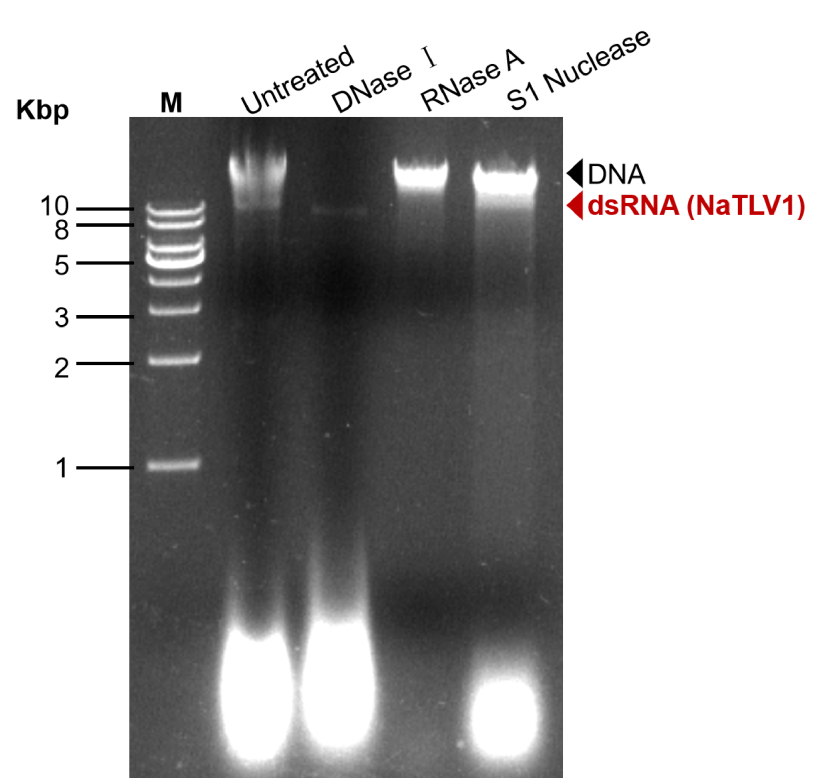


**FIG S1** Electrophoresis analysis and enzyme treatment of the dsRNA segment extracted from mycelia of *N. aurantiaca* strain A4. The electrophoretic profile on a 1.0% agarose gel of dsRNA preparations from NaTLV1 was extracted from the *N. aurantiaca* strain A4 following digestion with DNase I, RNase A, or S1 nuclease, and the untreated group.

**
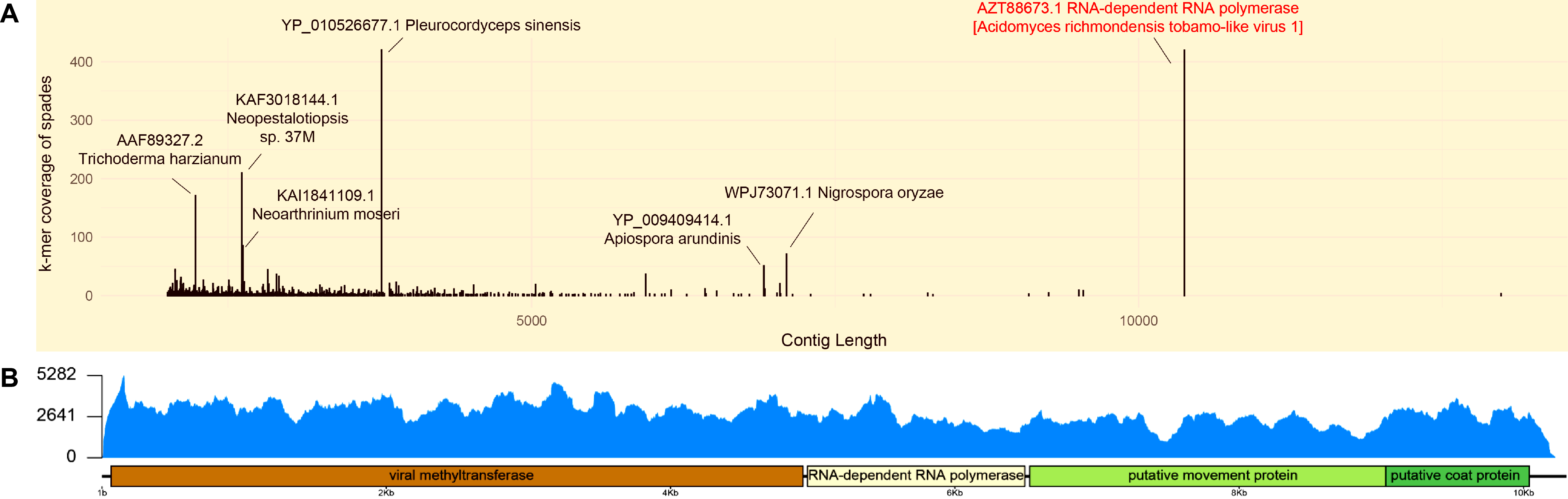
**

**FIG S2** Analysis of Illumina high-throughput RNA sequencing data based on total RNA extracted from the NaTLV1-infected (A4) *N. aurantiaca* strain. (A) The k-mer coverage of contigs longer than 2 kb obtained from SPAdes. The blastx best hit virus was marked in red. (B) Coverage of NaTLV1 reads through Bowtie2.


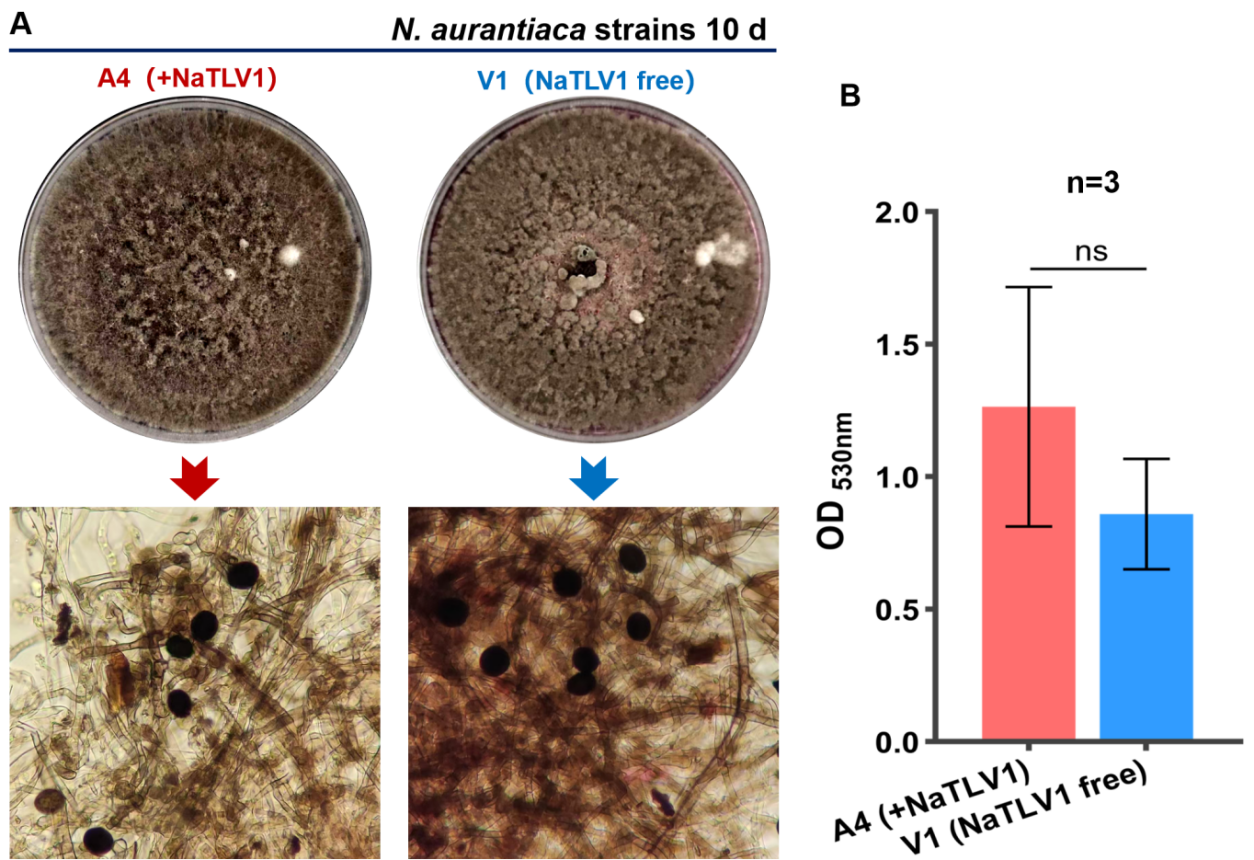


**FIG S3** Comparison of conidia morphology and production of the NaTLV1-infected (A4) strain and the NaTLV1-free (V1) strain. (A) Colony and conidia morphology of NaTLV1-infected (A4) strain and NaTLV1-free (V1) strain at 10 days. (B) The absorbance at 530 nm of the conidial suspension harvested from each plate that aforementioned the *N. aurantiaca* strain in (A). Error bars represent mean ± SD. *P < 0.05; ns, P > 0.05 (Student’s *t* test).

**
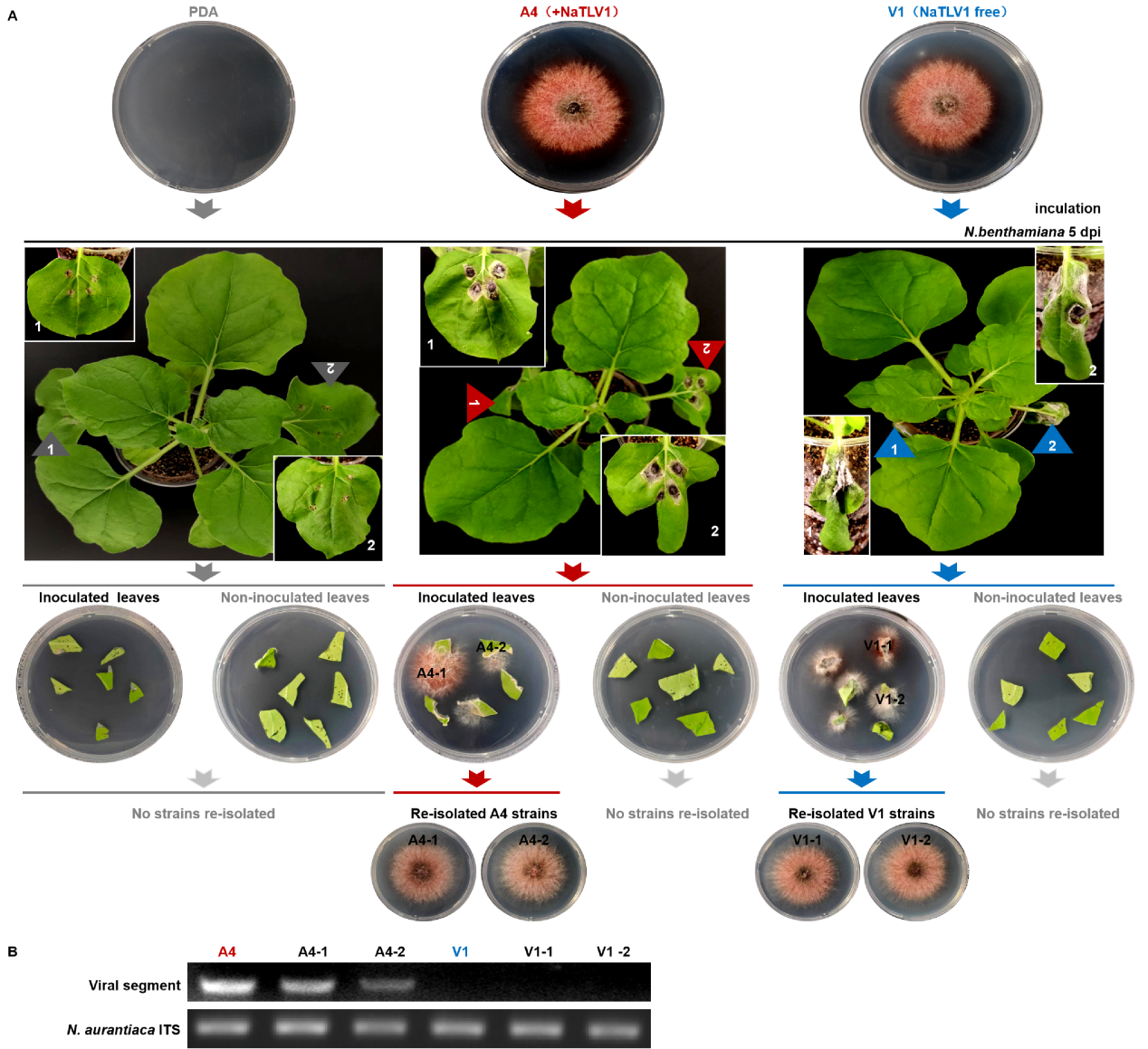
**

**FIG S4** Inoculation experiments involving NaTLV1-infected (A4) or NaTLV1-free (V1) *N. aurantiaca* strain on *N. benthamiana* leaves. (A) Representative symptoms (at 5 dpi) on *N. benthamiana* leaves following inoculation with NaTLV1-infected or NaTLV1-free strain. PDA indicates the negative control inoculated with uncolonized PDA disks. Insets: Close-up views of the inoculated leaves in (A). In addition, *N. aurantiaca*-inoculated and non-inoculated leaf tissues were placed on PDA medium to assess the presence of *N. aurantiaca* strains in the tissues. *N. aurantiaca* grew on the former but not on the latter. The fungi were photographed at 3 days after culturing. (B) Agarose gel electrophoresis of amplicons following RT-PCR (with oligonucleotide primers specific for NaTLV1 RNA) of the extracted RNA.

**
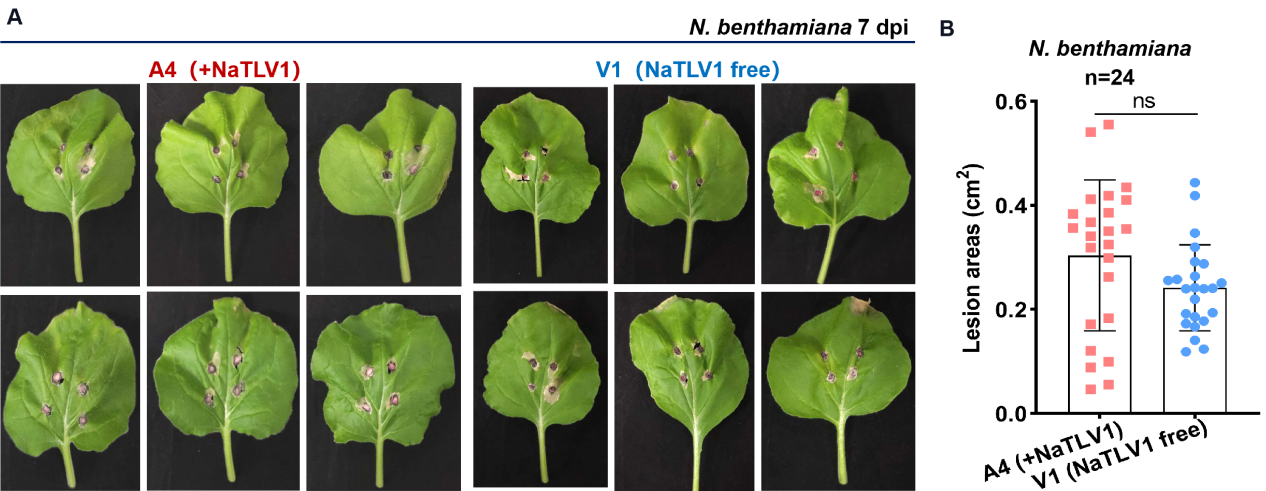
**

**FIG S5** virulence of NaTLV1-infected (A4) *N. aurantiaca* strain on *N.benthamiana* leaves. (A) Photographs of representative symptoms on N. *benthamiana* leaves at 7 dpi, inoculated with NaTLV1-infected (A4) and NaTLV1-free (V1) *N. aurantiaca* strain. (B) Lesion areas induced by inoculation with the aforementioned *N. aurantiaca* strain in (A). Error bars represent the standard deviation (SD). Red squares indicate individual measurements of inoculation sites for the A4 strain, and blue dots indicate individual measurements of inoculation sites for the V1 strain. ns, *P* > 0.05 (Student’s *t* test).


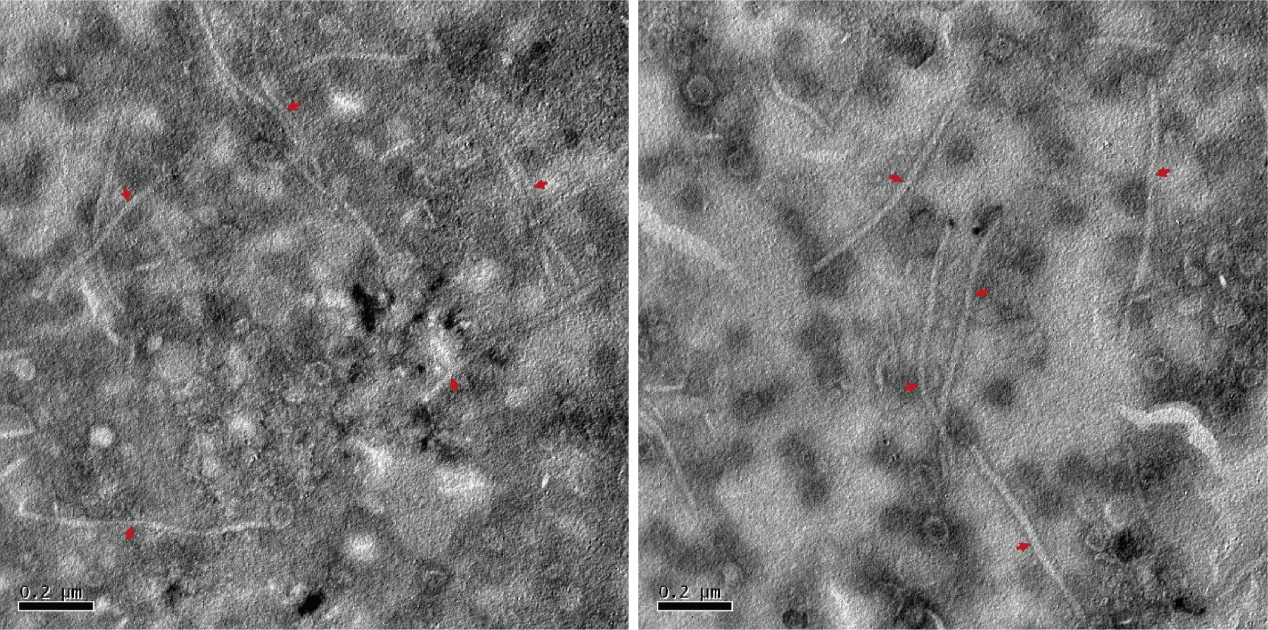


**FIG S6** Electron micrographs of the pellet of the fraction containing purified NaTLV1 (fraction 3) from NaTLV1-infected (A4) *N. aurantiaca* strain. Red arrowheads mark the potential NaTLV1 virus particles. Scale bar = 0.2 µm.


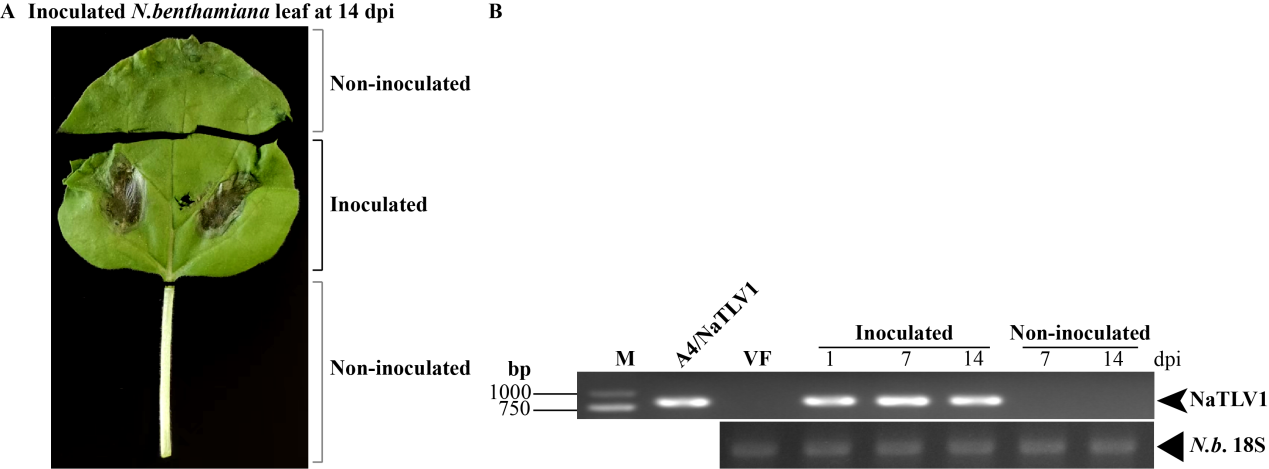


**FIG S7** NaTLV1 virus particles inoculated on an *N. benthamiana* leaf. (A) Photograph (at 14 dpi) of NaTLV1-related symptoms on an *N. benthamiana* leaf inoculated with NaTLV1 virus particles by mechanical friction inoculation. (B) Agarose gel electrophoresis image of the RT-PCR-based detection of NaTLV1 accumulation in inoculated and non-inoculated regions of a single *N. benthamiana* leaf, as described in (A). VF, virus free; N.b., *N. benthamiana.*
